# *Cryptococcus neoformans* phospholipase B1 is critical for cryptococcoma formation in the mouse brain

**DOI:** 10.3389/fimmu.2025.1681033

**Published:** 2025-10-15

**Authors:** Melissa E. Munzen, Glauber R. de Sousa Araújo, Mohamed F. Hamed, Marta Reguera-Gomez, Hiu Ham Lee, Bruno Pontes, Karina Alviña, Susana Frases, Luis R. Martinez

**Affiliations:** ^1^ Department of Oral Biology, University of Florida College of Dentistry, Gainesville, FL, United States; ^2^ Laboratório de Biofísica de Fungos, Instituto de Biofísica Carlos Chagas Filho, Universidade Federal do Rio de Janeiro, Rio de Janeiro, Brazil; ^3^ Department of Pathology, Faculty of Veterinary Medicine, Mansoura University, Mansoura, Egypt; ^4^ Department of Biomedical Sciences, NYIT College of Osteopathic Medicine, New York Institute of Technology, Old Westbury, NY, United States; ^5^ Laboratório de Pinças Ópticas, Instituto de Ciências Biomédicas Universidade Federal do Rio de Janeiro, Rio de Janeiro, Brazil; ^6^ Department of Neuroscience, College of Medicine, University of Florida, Gainesville, FL, United States; ^7^ Emerging Pathogens Institute, University of Florida, Gainesville, FL, United States; ^8^ Center for Immunology and Transplantation, University of Florida, Gainesville, FL, United States; ^9^ McKnight Brain Institute, University of Florida, Gainesville, FL, United States; ^10^ Center for Translational Research in Neurodegenerative Disease, University of Florida, Gainesville, FL, United States

**Keywords:** adhesion, capsule, cerebral cryptococcosis, cryptococcoma, *Cryptococcus neoformans*, fungal biofilms, phospholipase, polysaccharide

## Abstract

**Background:**

*Cryptococcus neoformans (Cn)* is an encapsulated, neurotrophic fungus that can cause life-threatening meningoencephalitis in immunocompromised individuals. Phospholipase B1 (PLB1) promotes *Cn* adhesion and colonization, however its role in fungal biofilm formation is not entirely clear.

**Methods:**

We investigated how PLB1 is involved in *Cn* infection using a stereotaxic intracerebral infection mouse model, microscopy, whole-genome sequencing, and biophysical methods.

**Results:**

Our results showed that the *PLB1-*disrupted strain exhibited reduced survival and capsular polysaccharide (CPS) dissemination throughout brain tissue and elicited a stronger microglial response *in vivo*. Moreover, *Cn* adhesion to SH-SY5Y human neuroblastoma cells was weakened in the *PLB1-*disrupted strain, and *in vitro* biofilm formation showed reduced metabolic activity and thickness. Both the *PLB1-*disrupted and -reconstituted strains showed structural alterations; nevertheless, CPS production was increased in the *PLB1*-disrupted cells. We show that PLB1 is essential for maintaining capsular elasticity, regulating CPS secretion, and biofilm formation, which are critical for fungal colonization and cryptococcoma formation.

**Conclusion:**

These results emphasize the need for further investigation into the mechanisms underlying the pathogenicity of *Cn*. In addition, our findings provide further evidence to validate PLB1 as an important antifungal target.

## Introduction

*Cryptococcus neoformans* (*Cn*) is an encapsulated yeast-like fungus that can cause life-threatening meningoencephalitis (CME) especially in immunocompromised individuals. The mortality rate of *Cn* infection is high (~74%), accounting for 112,000 annual deaths worldwide (1). While the infection is typically established in the pulmonary tract following inhalation of fungal spores, *Cn* dissemination into the bloodstream demonstrates the tropism of *Cn* for the central nervous system (CNS), where it causes extensive brain tissue destruction (2, 3). The polysaccharide capsule is a major contributor to *Cn* virulence (4). The capsule’s main constituent is glucuronoxylomannan (GXM), which accumulates in the serum and cerebrospinal fluid (4, 5) and contributes to *Cn* pathogenesis (6). High GXM levels are associated with many immunosuppressive effects (6), including interference with phagocytosis, antigen presentation, leukocyte migration and proliferation, and specific antibody (Ab) responses. GXM even enhances HIV replication (7).

Uncontrolled *Cn* infection can lead to the formation of localized biofilm-like lesions in the CNS known as cryptococcomas, a collection of yeast cells entangled in capsular material and characterized by adjacent neuronal loss. The formation of cryptococcomas in the CNS provides a plausible explanation for *Cn*’s successful neurotropism. This lesion, often surrounded by microglia (8), induces an innate immune response typically observed in infected individuals unable to fight the fungal burden. Instead of the microbicidal response, the cryptococcoma is encased with lymphocytes, macrophages, and multinuclear giant cells, resembling a chronic granulomatous reaction (9). The prognosis of patients with signs of suspected cryptococcoma depends on the location of the lesion. For example, lesions in the basal ganglia, brainstem, and cerebellum, the most common sites of cryptococcal involvement identified on neuroimaging (10, 11), result in signs and symptoms associated with CME, including headache, seizures, altered mental state, focal neurological defect, or blurred vision due to raised intracranial pressure. In fact, altered mental status is the most important predictor of poor outcome in CME patients (12, 13).

*Cn* phospholipase B1 (PLB1) is a virulence factor conveniently located in the cell wall via glycosylphosphatidylinositol (GPI) anchoring and can be secreted immediately in response to changing environmental conditions (14). The *PLB1* gene encodes for phospholipase B, lysophospholipase hydrolase, and phospholipase transacylase activities (15), which have been linked to destabilization of membranes, cell lysis, lipid signaling, immune evasion (16), and dissemination from pulmonary tissue into the bloodstream (15), exacerbating disease progression (17). Importantly, PLB1 activity is involved in fungal adherence to lung epithelial cells (18), as well as traversal of the blood-brain barrier (BBB) through Rac1 activation in endothelial cells (19), although it is not required for access to the brain tissue (20). The involvement of PLB1 in cryptococcoma formation is not fully understood, however its role in promoting fungal adherence to host cells and its importance for *Cn* proliferation suggests this enzyme is necessary for effective CNS colonization (21). Furthermore, deletion of the *PLB1* gene has demonstrated reduced virulence and proliferation in mouse infection models (16, 17, 21).

We recently established the importance of PLB1 in establishing cryptococcal infection and modulating the immune response in the CNS using a systemic mouse model of infection (21). Here, we investigate the implications of PLB1 in facilitating *Cn* colonization and cryptococcoma formation to augment the severity of CNS infection *in vivo*. To understand the role of PLB1 in the progression of cerebral cryptococcosis, we used a previously described stereotaxic CNS infection model (22) to directly inoculate C57BL/6 mice with wild-type H99, a *PLB1*-deletion (*plb1*) mutant, or a *PLB1* reconstituted or complemented (Rec1) strain (15). Our findings demonstrate that PLB1 facilitates *Cn* brain tissue colonization, promotes cryptococcoma formation, and supports capsular polysaccharide (CPS) synthesis and release both *in vivo* and *in vitro.* However, our observations of Rec1 suggest the involvement of additional regulatory or signaling components associated with *PLB1* which are necessary for PLB1 to confer effective virulence. These results validate our hypothesis that PLB1 activity contributes to *Cn* colonization of the CNS following intravenous infection and present PLB1 as a potential target for preventive antifungal therapy or disease management treatments, likely in combination with antifungal agents or additional therapeutic strategies, to improve survival outcome in patients afflicted with cerebral cryptococcosis.

## Results

### *Cn* strains *plb1* and Rec1 show prolonged survival in C57BL/6 mice after infection in the basal ganglia

**Figure 1:**
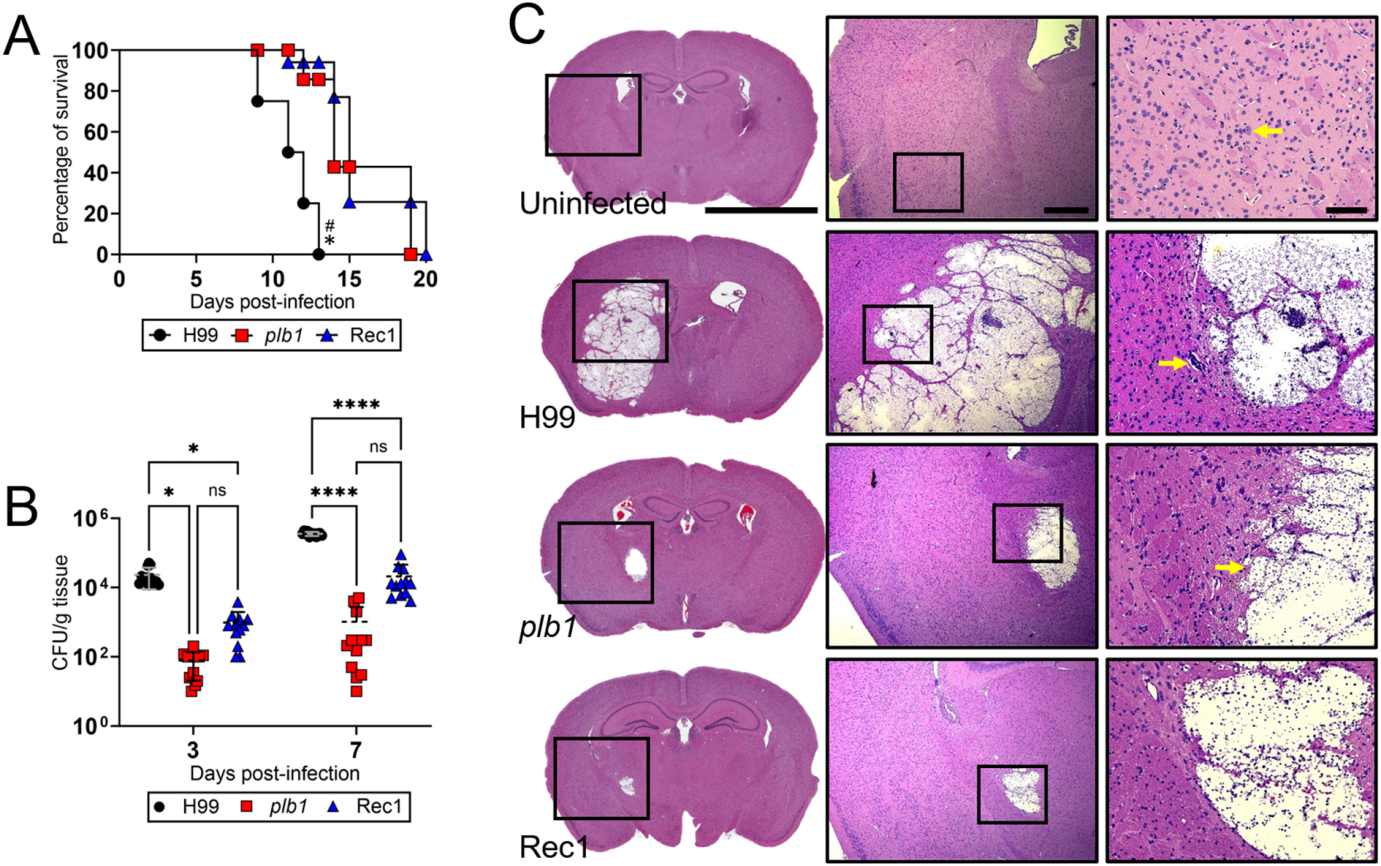
*C. neoformans* (*Cn*) strains *plb1* and Rec 1 show defective central nervous system (CNS) virulence in C57BL/6 mice infected in the basal ganglia. **(A)** Survival differences of C57BL/6 mice (*n* = 7 per group) intracerebrally (i.c.) infected with 10^4^ *Cn* strains H99 (median survival: 11.5-dpi), *plb1* (median survival: 14-dpi), or Rec1 (median survival: 15-dpi). *P* value significance (*P* < 0.05) was calculated by log rank (Mantel-Cox) analysis. Asterisk (*) and number (#) symbols denote higher mortality than *plb1*- and Rec1-infected animals, respectively. **(B)** Fungal burdens (colony forming units; CFU) in brains collected from *Cn* H99-, *plb1*-, or Rec1-infected mice with 10^4^ cryptococci (*n* = 12 per group) at 3- and 7-days post-infection (dpi). Bars and error bars denote the means and standard deviations (SDs), respectively. Asterisks denote *P* value significance (****, *P* < 0.0001; *, *P* < 0.05) calculated by analysis of variance (ANOVA) and adjusted using Tukey’s *post hoc* analysis. ns denotes comparisons that are not statistically significant. **(C)** Histological examinations from brains removed from *Cn* H99-, *plb1*-, or Rec1-infected mice with 10^4^ cryptococci at 7-dpi. Representative ×4 (left panel; scale bar, 200 μm), 10 (center panel; scale bar, 100 μm), and 20 (right panel; scale bar, 50 μm) magnifications of Hematoxylin and eosin-stained sections of the brain are shown. Black rectangular boxes delineate the area magnified (left to right panels). Arrows in the 20X magnification images indicate a specific histological finding explained in the result section.

To investigate the importance of PLB1 in *Cn* CNS pathogenesis, we intracerebrally (i.c.)-infected C57BL/6 mice with wild-type H99, *plb1*, or Rec1 strains. Mice infected with *plb1* (median survival: 14-dpi; *P* < 0.05) and Rec1 (median survival: 15-dpi; *P* < 0.05) strains exhibited similar prolonged survivability compared with H99-infected mice (median survival: 11.5-dpi; **Figure 1A**). Fungal burden was assessed in brain tissue at 3- and 7-dpi (*n* = 12 mice per group; **Figure 1B**). Brains infected with *plb1* (3-dpi: 7.8 x 10^1^ CFU/g tissue, *P* < 0.05; 7-dpi: 1.03 x 10^3^ CFU/g tissue, *P* < 0.0001) and Rec1 (3-dpi: 9.75 x 10^2^ CFU/g tissue, *P* < 0.05; 7-dpi: 2.1 x 10^4^ CFU/g tissue, *P* < 0.0001) revealed low fungal burden at both 3- and 7-dpi relative to H99 (3-dpi: 2.33 x 10^4^ CFU/g tissue; 7-dpi: 3.57 x 10^5^ CFU/g tissue).

Coronal hematoxylin and eosin (H&E)-stained brain sections from mice at 7-dpi confirmed the reduced cryptococcoma formation and disease severity in *plb1*- and Rec1-infected mice (**Figure 1C**), demonstrating potential impairment in the regulation or localization of the reinserted *PLB1* gene in Rec1. Higher magnification images demonstrated absence of inflammation around liquefactive necrosis, or regions with cellular structural loss, eosinophilia, and enucleation, and intralesional cryptococcal growth, as determined by fewer basophilic cells around the cryptococcal perimeter. *plb1*-infected brain tissue presented swollen endothelial cell lining of the blood capillary with perivascular edema (yellow arrow in **Figure 1C**), as characterized by enlarged endothelial cells and widened, less densely packed perivascular tissue. In contrast, H99-infected brain showed extensive cryptococcoma formation in the basal ganglia region that extended vertically to the striatum (e.g., putamen, globus pallidus), caudate nucleus, corpus callosum, and cortex, and laterally from the external to the internal capsule, nucleus accumbens, and anterior commissure. A high magnification image evinced minimal inflammation in the edges of the cryptococcoma, a large number of cryptococci, and a foreign body giant cell (yellow arrow in **Figure 1C**). Uninfected tissue exhibited normal neuroanatomic features in the basal ganglia region. High magnification images show normal blood vessels, glia, and dopaminergic neurons that are characterized by a large cell body with abundant basophilic cytoplasm and a vacuolated nucleus with a prominent central nucleolus (yellow arrow in **Figure 1C**). Together, our observations demonstrate that PLB1 is involved in *Cn* colonization of the CNS, conceivably in combination with other regulatory factors.

### Whole-genome sequencing reveals expected disruption and reinsertion of the *PLB1* gene in the *plb1* and Rec1 strains, respectively

**Figure 2:**
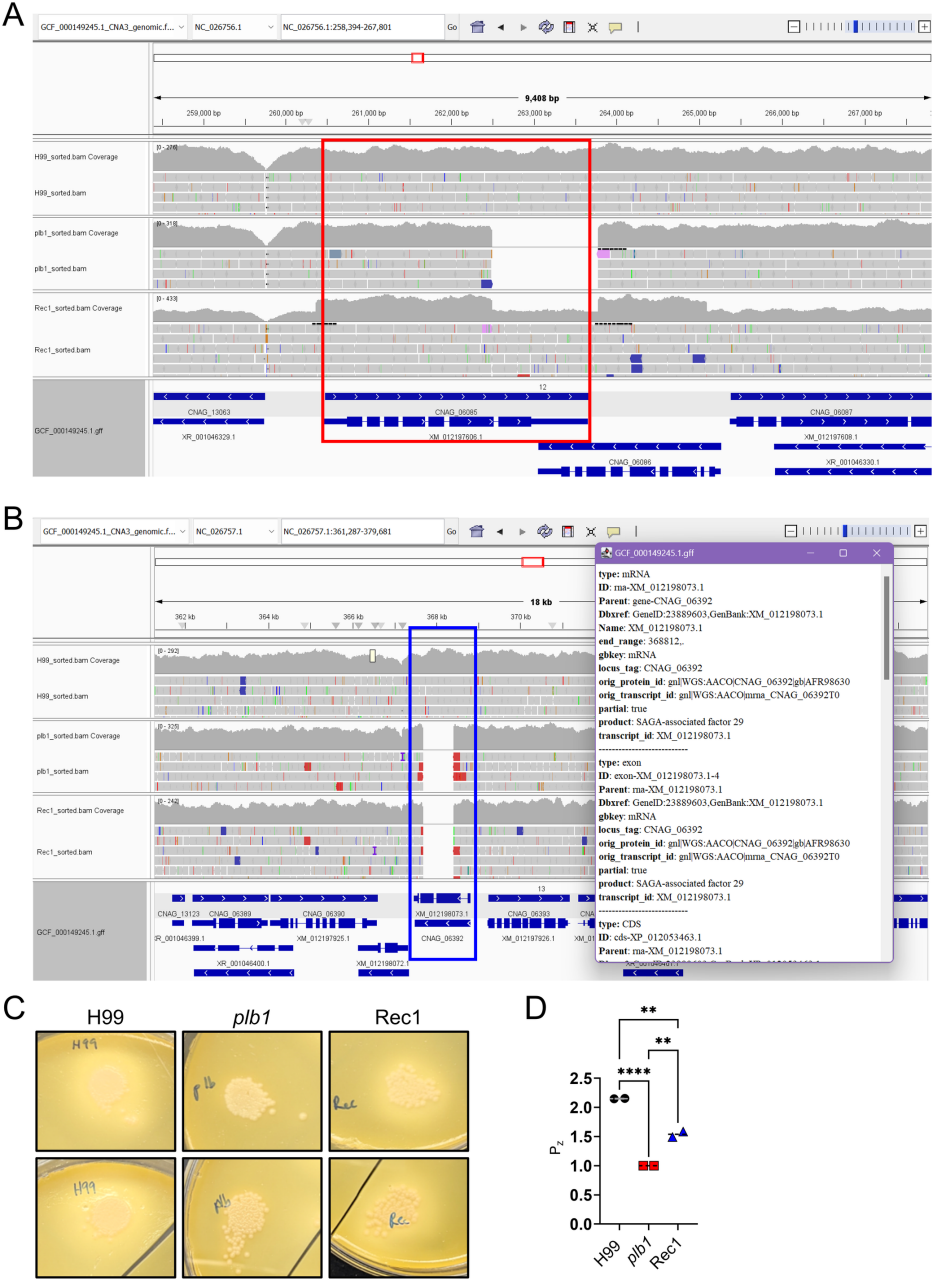
Whole-genome sequencing of *Cn* strains *plb1* and Rec 1 shows disruption construct and reinsertion of the *PLB1* gene in Rec1. **(A)** Genomes were sequenced and aligned to the reference *Cn* (RefSeq; GCF_000149245.1_CNA3) genome and observed in Integrative Genome Viewer (IGC, v. 2.18.2). The *PLB1* gene is outlined in red, indicating the presence of the gene in both H99 and Rec1 in chromosome 12. Part of (CNAG_06086) is deleted as part of the disruption construct in both *plb1* and Rec1, confirming accurate deletion of the *plb1* gene. **(B)** Disruption of SAGA-associated factor-29 (*SGF29*; CNAG_06392, blue outline) is observed in chromosome 13, supporting possible phenotypic differences between Rec1 and H99. **(C)** *Cn plb1* and Rec 1 strains show defective and restored phospholipase activity, respectively, in egg yolk agar. Colony growth of 5-µL 10^7^ *Cn* strains H99 (left panels), *plb1* (middle panels), or Rec1 (right panels) on egg yolk agar after 7 days. Precipitation or halo zones were observed around H99 and Rec1 strains. In contrast, there was a lack of phospholipase activity around *plb1* zone of growth. **(D)** Precipitation zone (Pz) was measured using FIJI ImageJ and calculated as Pz = (DC + PZ)/DC; DC = Diameter of Colony, PZ = Precipitation Zone. Dashed lines and error bars denote the means and SDs, respectively. Asterisks denote *P* value significance (****, *P* < 0.0001; **, *P* < 0.01) calculated by ANOVA and adjusted using Tukey’s *post hoc* analysis. Each strain was evaluated in duplicate colonies, and similar results were obtained as shown.

Our observations raised interesting questions about the impact of the *PLB1* gene insertion site within the Rec1 strain genome and the genetic regulatory factors of *PLB1* expression that may have affected the reconstituted strain’s ability to effectively regulate *PLB1* expression *in vivo*, which impaired cryptococcoma formation after direct CNS inoculation. To further examine the genomic distinctions between Rec1 and H99, we performed Next-Generation sequencing of the whole genomes of each strain (**Figure 2**). Following sequencing and alignment of each genome to the reference *C. neoformans* H99 var. *grubii* genome (RefSeq; GCF_000149245.1_CNA3), we utilized Integrative Genome Viewer (IGV) to view differences between the three genomes in parallel. We confirmed the disruption of the *PLB1* gene (CNAG_06085) and disruption construct of the neighboring cyclin-dependent kinase (CNAG_06086) in chromosome 12 of both *plb1* and Rec1 (**Figure 2A**), with Rec1 containing the reinserted gene (**Figure 2A**, red outline). Interestingly, chromosome 13 displayed a significant deletion within the gene encoding SAGA-associated factor 29 (*SGF29*; CNAG_06392) in *plb1* and Rec1, but not H99 (**Figure 2B**, blue outline). Mutations in this gene result in reduced histone acetylation and may promote hypervirulence, as reported in clinical strains (23). This activity may be dependent on the presence of the *PLB1* gene, which may explain why *plb1* is deficient in virulence compared with Rec1. The potential disruption of *SGF29* and its contribution to virulence in Rec1 compared with *plb1* and H99 are necessary to explore further in the context of both *SGF29*- and *PLB1-*associated virulence, as the disruption of *SGF29* or the dysregulation of *PLB1* or related signaling pathways following genetic manipulation may contribute to the inconsistencies in Rec1 behavior.

To complement the genomic data and verify the lack of phospholipase activity in the *plb1* strain, an egg yolk emulsion assay was performed in duplicates (**Figure 2C**; top and bottom panels). We observed obvious lack of precipitation or halo zone around the independent *plb1* strain colonies (middle panels) compared with H99 (left panels) and Rec1 (right panels) strains. Moreover, the Pz value is a measure of phospholipase activity obtained by calculating the ratio of the total diameter of the colony including the precipitation zone to the diameter of the colony. The Pz values were calculated for H99, *plb1*, and Rec1 strains using FIJI ImageJ software (**Figure 2D**). H99 showed the highest level of phospholipase activity (mean Pz value = 2.152) compared with Rec1 (mean Pz value = 1.540; *P* < 0.01) and *plb1* (mean Pz value = 1; *P* < 0.0001). The Pz value of *plb1* was 1.0, indicating no phospholipase activity present. This phenotypic confirmation supports the production of PLB1 by wild-type H99, the lack of phospholipase activity in *plb1*, and the restoration of the PLB1 phenotype in Rec1.

### *plb1* strain released less GXM and reduced microglia recruitment in brain tissue

**Figure 3:**
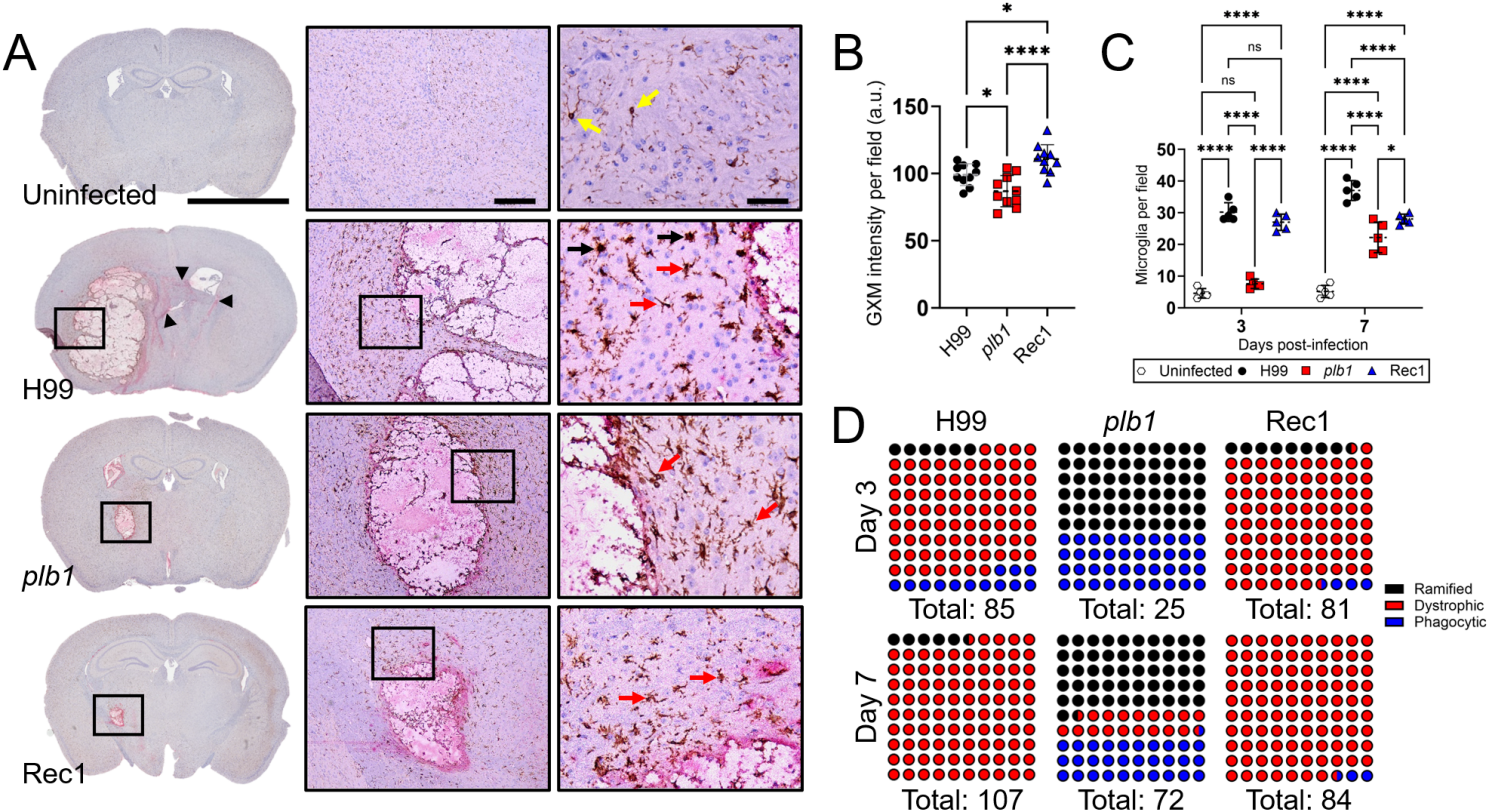
*Cn plb1* strain cells demonstrate reduced glucuronoxylomannan (GXM) release in brain tissue. **(A)** Histological examinations of the basal ganglia from brains removed from *Cn* H99-, *plb1*-, or Rec1-infected mice with 10^4^ cryptococci at 7-dpi. Representative 4X (left panel; scale bar, 200 μm), 10 (center panel; scale bar, 100 μm), and 20 (right panel; scale bar, 50 μm) magnifications of ionized calcium binding adaptor protein (Iba-1; microglia) and GXM binding monoclonal antibody (mAb) 18B7-stained sections of the brain are shown. Black rectangular boxes delineate the area magnified (left to right panels). Brown and red-pink staining indicate microglia and GXM accumulation, respectively. Arrowheads in 4X magnification denote GXM dissemination distal to the brain lesion. Yellow, red, and black arrows in the 20X magnification point to ramified, dystrophic, and phagocytic microglia. **(B)** Quantification of GXM intensity. Regions of GXM release were measured. Each symbol represents GXM intensity per individual field (*n* = 10 per group). a.u. denotes arbitrary units. **(C)** Counts of microglia per field of cortical tissue excised from C57BL/6 mice infected with H99, *plb1*, or Rec1 after 3- and 7-days were determined using light microscopy. Each symbol represents the number of microglia per individual field (*n* = 5 per group). For B and C, dashed lines and error bars denote the means and SDs, respectively. Asterisks denote *P* value significance (****, *P* < 0.0001; *, *P* < 0.05) calculated by ANOVA and adjusted using Tukey’s *post hoc* analysis. ns denotes comparisons that are not statistically significant. **(D)** Microglial type abundance (%; each dot is equivalent to 1%) during cerebral cryptococcosis. Microglia in basal ganglia tissue of uninfected or *Cn* H99-, *plb1*-, or Rec1-infected mice were visualized under the microscope 3- and 7-dpi and classified according to their morphology as ramified (black), dystrophic (red), and phagocytic (blue).

To visualize fungal GXM release and microglia distribution, we immunostained coronal brain sections from mice at 7-dpi with GXM-binding monoclonal antibody 18B7 (mAb18B7; red-pink staining) and ionized calcium-binding adaptor protein (Iba-1)-binding mAb specific for microglia (brown staining in **Figure 3A**), respectively. *Cn* H99-infected brain tissue exhibited GXM distribution diffusing away from the edges of the cryptococcoma (arrowheads), and numerous aggregates of microglia in the periphery of the necrotizing lesion. High magnification images showed numerous microglia cells with pleomorphic morphology, including dystrophic (not-well defined soma and thin ramifications; red arrows) and phagocytic or amoeboid (large soma and short-retracted ramifications; ameboid or macrophage-like; black arrows) phenotypes. Brain tissue infected with *plb1* cryptococci showed GXM intensity limited to the inside and edges of the cryptococcoma, without significant tissue distribution. Microglial cells surrounding the *plb1* biofilm-like lesion were mostly ramified or homeostatic (cylindrical soma and long and thin ramifications; also known as branched; arrows), which typically maintain CNS homeostasis in apparently healthy brains (24). In addition, Rec1-infected brain tissue presents thick accumulation of GXM in the edges of the cryptococcoma and an arborizing smear that extends to neighboring tissue. Brown-stained microglia had mainly a dystrophic (arrows) morphology and accumulated around the edges of the Rec1-cryptococcoma. Uninfected brains show few scattered ramified microglia (yellow arrows) in tissue.

Analysis of the intensity of GXM staining (*n* = 10 fields per strain) in brain tissue excised from Rec1-infected mice revealed higher GXM distribution than in tissue infected with H99 (*P* < 0.05) or *plb1* (*P* < 0.0001) strains (**Figure 3B**). *plb1-*infected tissue exhibited a reduction in GXM release intensity compared with H99 (*P* < 0.05). Using a systemic *Cn* infection mouse model, we previously demonstrated that PLB1 alters microglia responses and morphology (21). To confirm the involvement of PLB1 in microglia recruitment upon *Cn* CNS infection, microglia were quantified in multiple fields surrounding the area of infection (**Figure 3C**). Interestingly, there was no difference in microglia numbers between *plb1*- and uninfected brain tissue at 3-dpi, while microglia numbers were substantially higher in the H99- (*P* < 0.0001 relative to uninfected and *plb1*) and Rec1- (*P* < 0.0001 relative to uninfected and *plb1*) infected tissue. There was no statistical difference in microglial recruitment between H99- and Rec1-infected brains. At 7-dpi, the microglia recruitment increased in *plb1*-infected tissue but remained lower than in H99- (*P <* 0.0001) and Rec1- (*P* < 0.05) infected tissue. Interestingly, microglia recruitment in brain tissue infected with Rec1 remains similar at 7-dpi, although significantly lower than in H99-infected tissue (*P* < 0.0001). Finally, microglial cells can present diverse morphological phenotypes in tissue during *Cn* CNS infection (21), and these distinct phenotypes have been associated with several neurodegenerative diseases (25, 26). *Cn* H99 (81.2%)- and Rec1 (87.7%)-brain infected tissue evinced similar microglia phenotype abundance at 3-dpi, with dystrophic cells (H99: 81.2%; Rec1: 87.7%) being the most prevalent phenotype, while ramified (60%) microglia were the dominant phenotype in *plb1*-infected brains (**Figure 3D**). Moreover, *plb1* had the highest proportion of phagocytic microglia at both 3 (60%)- and 7 (30.5%)-dpi. Notably, phagocytic microglia were completely and partially (25%) reduced in H99- and *plb1*-infected tissue, respectively, being replaced by dystrophic cells at 7-dpi (**Figure 3D**).

Taken together, our results indicate that microglial responses are influenced by PLB1 production and the amount of GXM released during infection.

### *Cn plb1* exhibits reduced biofilm formation

**Figure 4:**
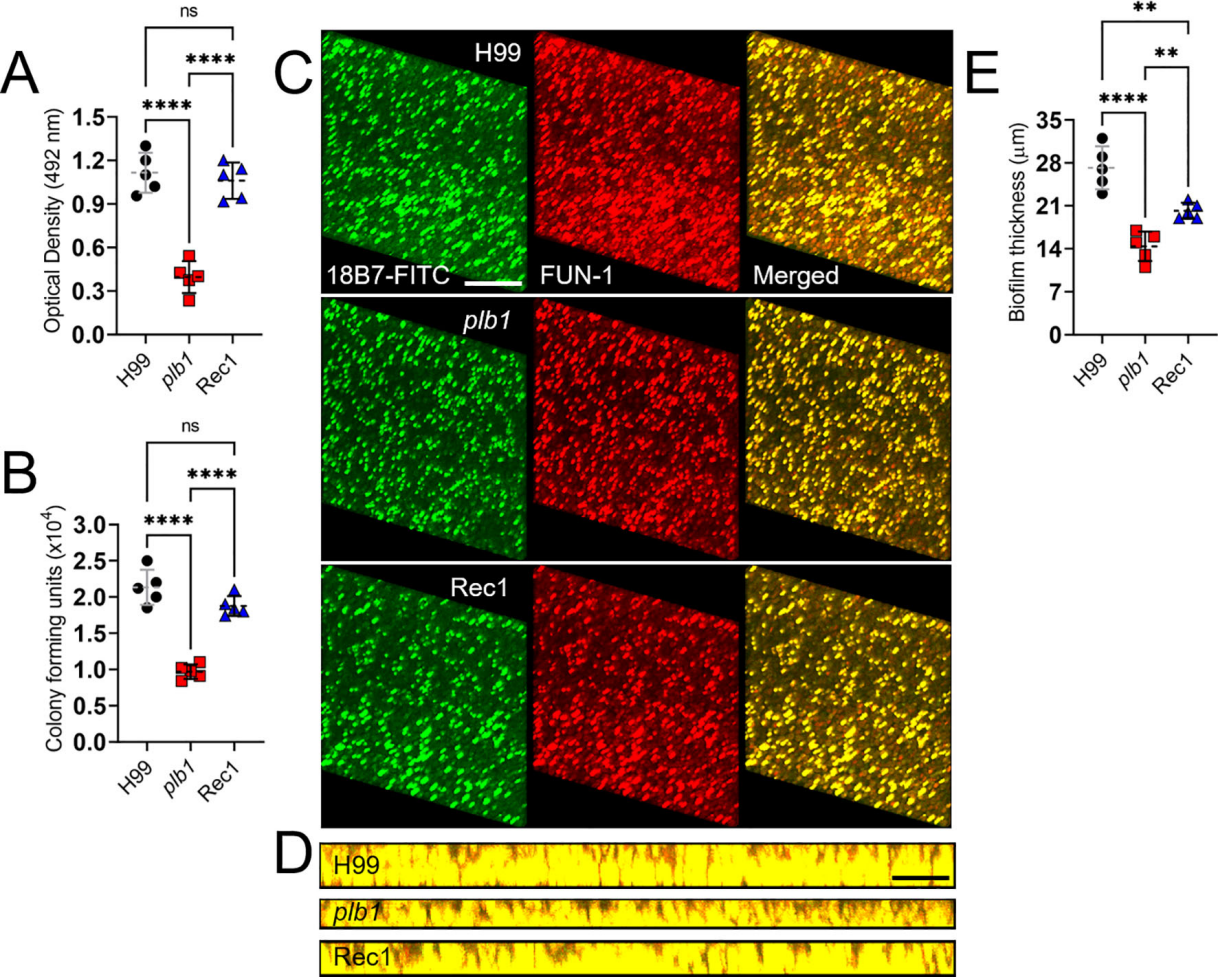
*Cn plb1* strain shows decreased biofilm formation *in vitro*. Biofilm formation by *Cn* strains H99, *plb1*, and Rec1 was compared and determined using **(A)** XTT reduction and **(B)** CFU determination assays. **(C)** Representative confocal microscopic images of *Cn* H99, *plb1*, and Rec1 strain biofilms after 48 h Representative images of mature fungal biofilms showed metabolically active (red; FUN-1-stained) cells embedded in the polysaccharide extracellular material (green; stained with mAb 18B7-FITC-conjugated GAM IgG1). **(D)** The thickness and morphology of the cryptococcal biofilms can be observed. For C and D, the pictures were taken at a magnification of 63X. Scale bars, 20 µm. **(E)** The thickness of the cryptococcal biofilms was determined using *Z*-stack reconstruction. For A, B, and E, dashed lines are the averages of the results for five independent measurements (each symbol represents an individual measurement) per strain, and error bars denote SDs. Asterisks denote *P* value significance (****, *P* < 0.0001; **, *P* < 0.01) calculated by ANOVA and adjusted using Tukey’s *post hoc* analysis. ns denotes comparisons that are not statistically significant.

*Cn* forms biofilms on polystyrene plates and medical devices including ventriculoatrial shunt catheters (27, 28). *Cn* GXM is extensively released, building up around attached cells and encasing them within an exopolymeric matrix (29). Since cryptococcomas are biofilm-like brain lesions, we investigated the impact of PLB1 in biofilm formation *in vitro* (**Figure 4**). Biofilm formation by *Cn* strains H99, *plb1*, and Rec1 was compared and determined using XTT reduction assay (**Figure 4A**), CFU determinations (**Figure 4B**), and confocal microscopy (**Figures 4C,D**). *Cn* strains H99 (*P* < 0.0001) and Rec1 (*P* < 0.0001) cryptococci showed higher metabolic activity (**Figure 4A**) and viability (**Figure 4B**) than *plb1* cryptococci. We used confocal microscopy to compare the XTT reduction assay and CFU determination findings with the properties of biofilm metabolism and architecture (**Figures 4C,D**), visualized by fluorescent binding to intravacuolar structures indicative of cryptococcal metabolic activity within the exopolymeric biofilm matrix. Regions of red fluorescence (FUN-1) represent metabolically active cells, and the green fluorescence [monoclonal antibody (mAb) 18B7-fluorescein-isothiocyanate (FITC)-conjugated GAM IgG_1_] indicates GXM. Cryptococci from each strain exhibited a uniform distribution and biofilm formation across the field, with high metabolic activity in yeasts surrounded by extracellular GXM material (**Figure 4C**). To visualize biofilm thickness, in-depth *Z*-stack reconstruction showed that on average, *Cn* H99 biofilms (27.2 µm; range: 23-32 µm) were thicker compared to *plb1* (14.4 µm; range: 11-17 µm; *P* < 0.0001) and Rec1 biofilms (20.2 µm; range: 19-22 µm; *P* < 0.01 relative to the other groups) (**Figures 4D,E**). These data confirm that *Cn* PLB1 or manipulation of the *PLB1* gene are critical for biofilm formation and purportedly cryptococcoma formation during infection.

### *Cn plb1* strain shows reduced adhesion to SH-SY5Y human neuroblastoma cells

**Figure 5:**
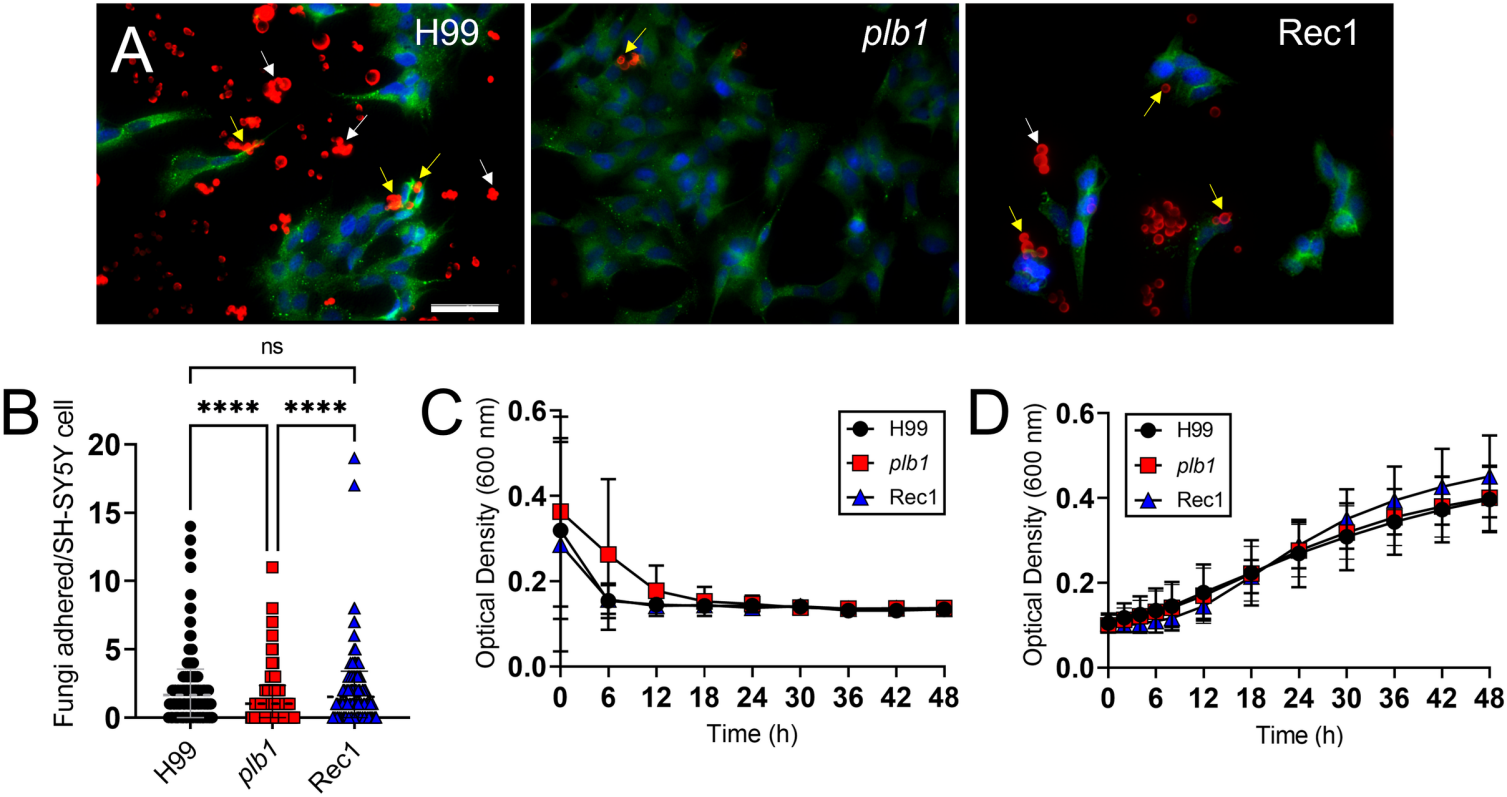
*Cn plb1* strain demonstrates less adhesion to SH-SY5Y human neuroblastoma cells. **(A)** Fluorescent images of *Cn* strain H99, *plb1*, and Rec1 cells interacting with SH-SY5Y human neuroblastoma cells. After 4 h co-incubation at 37 °C, SH-SY5Y human neuroblastoma cells were washed and incubated with DAPI and β-tubulin to label the nuclei (blue) and cell body (green), respectively. Cryptococci were incubated with mAb 18B7-rhodamine-conjugated goat anti-mouse IgG1 stained to label the capsular polysaccharide (CPS; red). Scale bar, 50 µm. **(B)** Adhesion of H99, *plb1*, and Rec1 cryptococci to SH-SY5Y cells was determined by counting using an inverted microscope. Dashed lines indicate the averages of the number of fungi attached to individual neuroblastoma cells (*n* = 100 per group), and error bars denote SDs. Asterisks denote *P* value significance (****, *P* < 0.0001) calculated by ANOVA and adjusted using Tukey’s *post hoc* analysis. ns denotes comparisons that are not statistically significant. Growth curve for *Cn* H99, *plb1*, and Rec1 grown in **(C)** neuroblastoma complete or **(D)** Sabouraud dextrose broth for 48 h at 37 °C. A Bioscreen C spectrophotometer was used to take each measurement in real time. Each time point represents the averages from 30 independent wells per time point, and error bars indicate SDs. These experiments were performed twice, and similar results were obtained.

*Cn* biofilm formation depends on the presence of the polysaccharide capsule (30), the interactions of cryptococci with host cells (31), and the ability of the CPS to bind the colonizing substrate (30). We investigated the importance of PLB1 in cryptococcal adhesion to and colonization of human neuroblastoma cell line SH-SY5Y [4′, 6-diamidino-2-phenylindole (DAPI), blue nuclei; β-tubulin, green cell body] using fluorescent microscopy (**Figure 5**). *Cn* strain H99 cells [GXM binding mAb 18B7-rhodamine-conjugated goat anti-mouse IgG1; red fungi] cultured with SH-SY5Y neuroblastoma cells revealed a considerable number of cryptococci bound either to the mammalian cells (yellow arrows) or the glass-bottom substrate (white arrows) as single cells or cell aggregates (**Figure 5A**; left panel). Neuroblastoma cells incubated with *plb1* cells displayed only a few cryptococci bound (center panel). Rec1 cells evinced uneven distribution but similar single or aggregate cell binding to neuroblastoma cells or the glass-bottom surface as observed in wild-type cells (**Figure 5A**; right panel), potentially due to impaired *PLB1* regulation or PLB1 anchoring to or secretion from the fungal membrane. Quantification of the adhesion ability of *Cn* strain H99 (*P* < 0.0001) and Rec1 (*P* < 0.0001) cells demonstrated increased adhesion to SH-SY5Y neuroblastoma cells relative to that of *plb1* (**Figure 5B**). Moreover, we performed a growth curve in neuroblastoma complete medium (**Figure 5C**) and Sabouraud dextrose broth (**Figure 5D**) using Bioscreen C analysis to compare the replication rates of *Cn* strains H99, *plb1*, and Rec1 to validate that the differences in adhesion and biofilm formation observed were not caused by defects in the mutant growth. We did not observe any differences in growth rates in either medium between the groups 48 h after culture inoculation. Therefore, these findings indicate that *Cn plb1* strain has reduced adhesion to SH-SY5Y human neuroblastoma cells, suggesting that PLB1 is involved in host cell colonization.

### *plb1* and Rec1 cryptococci display reduced CPS fibers interacting with a solid surface and local GXM release

**Figure 6:**
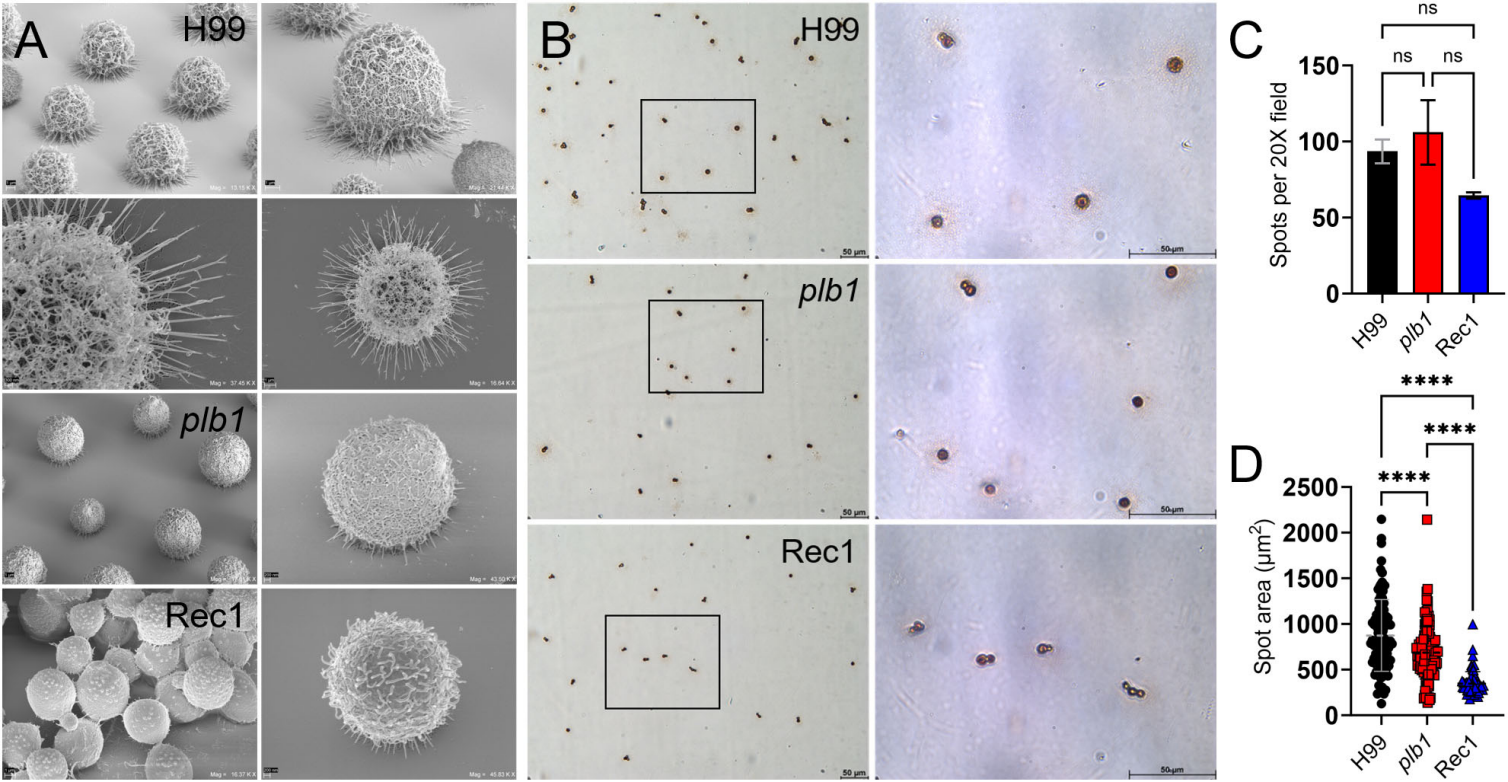
*Cn* strain H99 capsule displays longer fibers and increased capsular material released in the adhesion area compared to *plb1* and Rec1 strains. **(A)** Tilted scanning electron microscopy (SEM) images of *Cn* strains H99 (top 4 images), *plb1*, and Rec1 cells after 2 h adhesion to a solid surface. Panel magnifications (left to right) of representative images of the cryptococci and fibril extensions (left panel; scale bar, 1 μm, and right panel; scale bar, 200 nm). **(B)** Light microscopy images of spots formed by *Cn* strains H99, *plb1*, and Rec1 during 2 h incubation ELISA spot assay. Representative 20X (left panel; scale bar, 50 μm) and 100 (right panel; scale bar, 50 μm) magnifications are shown. Black rectangular boxes delineate the area magnified (left to right panels). **(C)** Spot number in microtiter wells for each cryptococcal strain. Bars are the average numbers of spots in six 20X power fields, and error bars denote SDs. **(D)** The average spot area was quantified using FIJI ImageJ software and calculated using area = πr^2^. The average (each symbol represents 1 spot; H99, *n* = 187; *plb1*, *n* = 212; and Rec1, *n* = 129) and SDs of the spot area for each strain are shown. For **(C, D)**, asterisks denote *P* value significance (****, *P* < 0.0001) calculated by ANOVA and adjusted using Tukey’s *post hoc* analysis. ns denotes comparisons that are not statistically significant. This experiment was performed twice with similar results.

*Cn* biofilm formation depends on CPS synthesis and release (30). Given that attachment to a surface initiates fungal biofilm formation (29), we evaluated the importance of PLB1 in this critical stage (**Figure 6**). We first performed tilt angle scanning electron microscopy (SEM) where the specimen was tilted 40 degrees to observe how H99, *plb1*, and Rec1 cells interact with the binding surface (**Figure 6A**). *Cn* H99 strain showed intertwined CPS throughout each cell, adhering strongly and homogeneously across the solid surface (upper left and right panel). Each H99 cryptococci displayed many long CPS fibers uniformly distributed (lower left and right panels) around the base of each cell in contact with the binding surface. In contrast, *plb1* cryptococci adhered across the solid surface with minimal encapsulation (left image) and short CPS fibers in the region interacting with the binding surface (right image). Likewise, Rec1 cells were aggregated (lower left) across the power field, and although they synthesized thicker CPS fibers than those observed on *plb1* cells, these fibers were short. The interaction of Rec1 cells with the binding surface was comparable to that observed in *plb1* cells (lower right).

To investigate the differences in adhesion, a critical step in biofilm formation, shown by H99, *plb1*, and Rec1 cells, we used an ELISA spot assay. We particularly focused on potential differences between the strains in local CPS released upon contact with the binding surface (**Figure 6B**). Light microscopy was used to quantify the number of cells attached to the solid surface as measured by spot formation (**Figure 6C**). While all the strains formed a comparable number of spots (**Figure 6C**), they differed significantly in the spot area, with the largest being H99 > *plb1* > Rec1 cells (*P* < 0.0001; **Figure 6D**). These results suggest that PLB1 is involved in local CPS release, but the reduced CPS release and elongation of CPS fibers in Rec1 potentiate that other factors regulating *PLB1* expression, production, or secretion may also be involved for proper *Cn* adhesion and biofilm formation.

### PLB1 is important in the distribution of cryptococcal CPS

**Figure 7:**
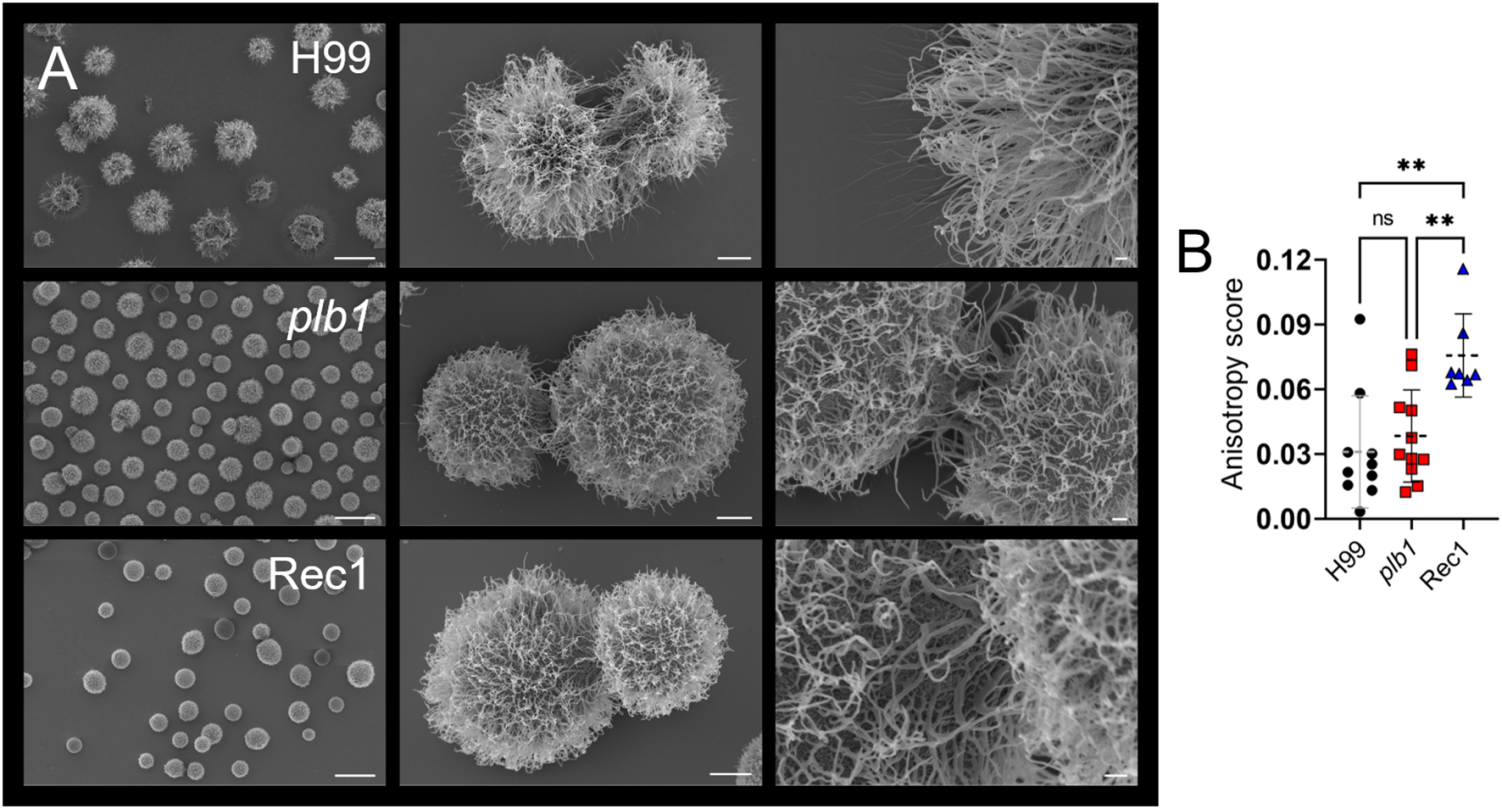
*Cn* strain Rec1 shows increased fibril array anisotropy relative to H99 and *plb1* strains. **(A)** SEM images of *Cn* strains H99, *plb1*, and Rec1 cells illustrate their morphology and capsular density (left panel; scale bar, 10 μm; central panel; scale bar, 1 μm, and right panel; scale bar, 200 nm). **(B)** The anisotropy score of polysaccharide fibers around single cryptococci from H99 (*n* = 10), *plb1* (*n* = 11), and Rec1 (*n* = 7). Each symbol indicates a single measurement. Dashed lines and error bars denote the means and SDs, respectively. Asterisks denote *P* value significance (**, *P* < 0.01) calculated by ANOVA and adjusted using Tukey’s *post hoc* analysis. ns denotes comparisons that are not statistically significant.

*Cn* dynamically produces and secretes its CPS, which enhances the ability of the fungus to adhere to surfaces and form biofilms (30). Scanning electron microscopy (SEM) images demonstrated that *plb1* and Rec1 yeast cells had similar sizes but smaller capsules than H99 cryptococci under normal growing conditions (**Figure 7A**). H99 cryptococci showed long CPS fiber extensions that protrude several microns from the cell body. In contrast, *plb1* and Rec1 cells exhibited smaller CPS fibers, and this fundamental difference may prevent fungal cell adhesion, which can play a critical role in host tissue colonization and cryptococcoma formation.

Since *plb1* and Rec1 cells showed reduced biofilm formation, local CPS release, and CPS fiber length compared to H99 cells, we measured the anisotropy of each strain’s CPS fiber arrays and their average orientation in *Cn* cells (32), directly from SEM images (**Figure 7B**). Anisotropy is a structural property that describes uniformity in different directions (i.e., order). Rec1 cells had the highest anisotropy score (*P* < 0.01), indicating the most ordered CPS fiber arrays. In contrast, H99 and *plb1* cells demonstrated no difference in anisotropy scores, indicating randomly oriented CPS fiber distribution. Taken together, these results suggest that *PLB1* expression is important for CPS distribution and fiber organization, although not solely responsible.

### *plb1* cells grow larger in volume and capsule size in response to capsular induction growth conditions

**Figure 8:**
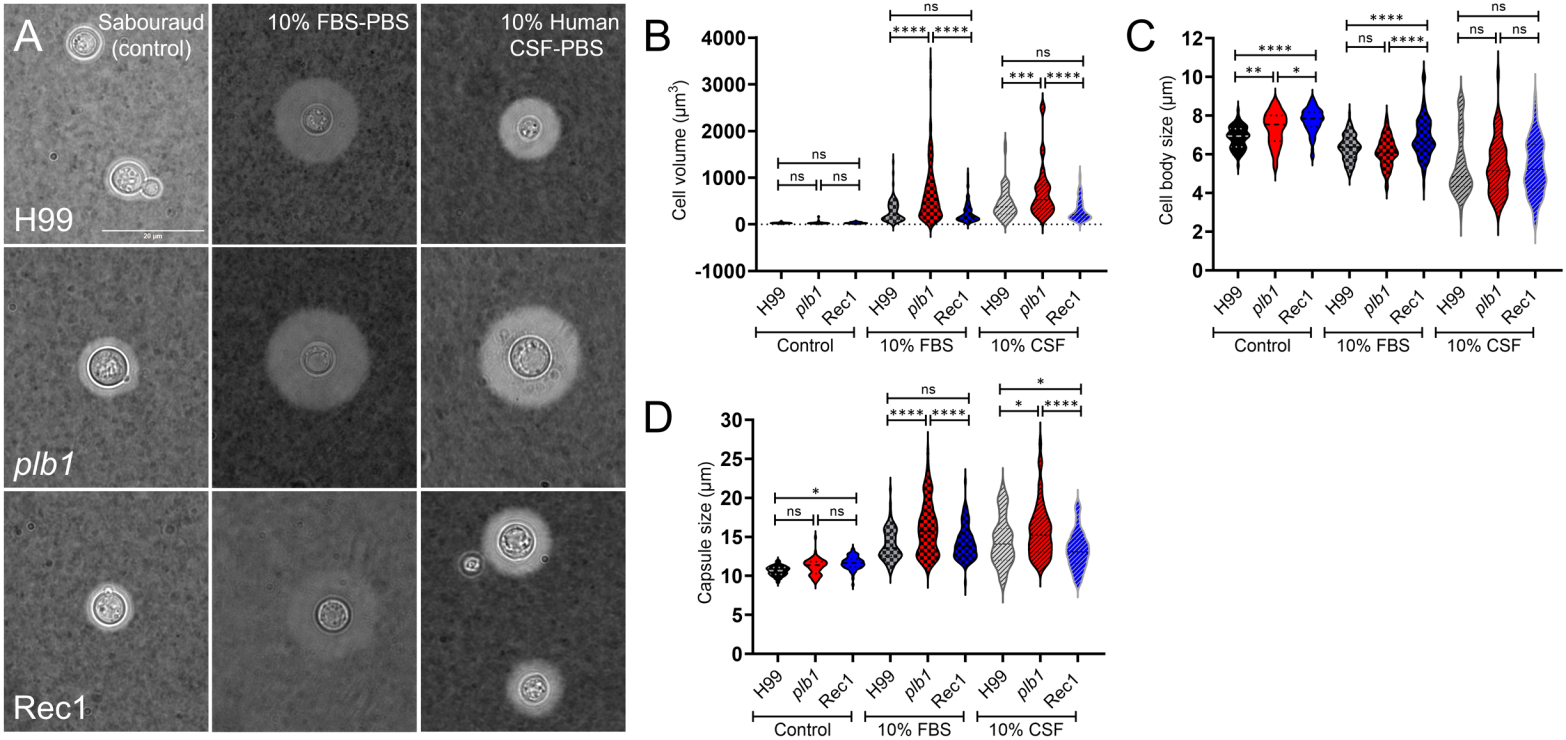
*Cn* strain *plb1* demonstrates increased cell volume and capsule size compared with H99 and Rec1 strains after growth in capsular induction conditions. **(A)** Representative light microscopy images of *Cn* strains H99, *plb1*, and Rec1 cells stained with India ink are shown. Cryptococci were grown in Sabouraud dextrose broth (control; left panels) or phosphate-buffered saline (PBS) supplemented with 10% fetal bovine serum (FBS; middle panels) or human cerebrospinal fluid (CSF; right panels) at 37 °C in an atmosphere containing 10% CO_2_ for 24 h The pictures were taken using a 100X power field. Scale bar: 20 µm. **(B)** Cell volume (V = 4/3πr^3^), **(C)** cell body size (whole cell diameter – capsule diameter), and **(D)** capsule size (whole cell diameter – cell body diameter) of H99, *plb1*, and Rec1 strains were measured. For B-D, violin plots show the averages (*n* ≥ 55–90 cells per group; dashed lines) and SDs of the results. Asterisks denote *P* value significance (****, *P* < 0.0001; ***, *P* < 0.001; **, *P* < 0.01; *, *P* < 0.05; ns, not significant) calculated by ANOVA and adjusted using Tukey’s *post hoc* analysis.

*Cn* has been shown to increase capsule size when exposed to serum (33) and cerebrospinal fluid (CSF (34);). Capsular enlargement occurs during infection and results in reduced phagocytosis by host immune cells (35), serving as an important virulence factor. To evaluate the involvement of PLB1 in *Cn* capsular growth response, we grew H99, *plb1*, and Rec1 cultures in Sabouraud dextrose broth (control) or phosphate-buffered saline (PBS) supplemented with 10% fetal bovine serum (FBS) or human cerebrospinal fluid (CSF; capsular induction medium) at 37 °C overnight (**Figure 8**). Light microscopy imaging using India ink staining showed that while cryptococci from all strains undergo capsular enlargement compared with cells grown in normal conditions (**Figure 8A**), the capsules (white halo) of *plb1* cells were larger than those of H99 and Rec1 cells.

We analyzed the cell volume (**Figure 8B**), body size (**Figure 8C**), and capsule size (**Figure 8D**) of each strain after growth in control and capsular inducing medium. There were no differences in cell volume among the strains grown in normal conditions (**Figure 8B**). Interestingly, under capsule induction conditions with 10% FBS or CSF (**Figure 8C**), cryptococci of each strain had significantly smaller cell body size than those cultured under normal conditions. However, under normal conditions, we observed a significant increase in the cell body size of Rec1 (mean: 7.75 μm) compared to H99 (mean: 6.87 μm; *P* < 0.0001) and *plb1* (mean: 7.35 μm; *P* < 0.05) strains (**Figure 8C** **, control**). The cell body size of *plb1* was also significantly larger than those seen in H99 cells (*P* < 0.01; **Figure 8C**, control). In addition, the capsule size of Rec1 (mean: 11.67 μm) under normal conditions was larger than those of H99 cells (mean: 10.65 μm; *P* < 0.05; **Figure 8D**, control), although no differences were observed when these strains were compared to *plb1* cells (mean: 11.19 μm).

Under capsule induction conditions, *plb1* (FBS, mean: 668.5 μm^3^ and 16.11 μm; CSF, mean: 697.4 μm^3^ and 15.76 μm) exhibited a substantial cell volume enlargement (**Figure 8B**) and capsule size (**Figure 8D**) relative to H99 (FBS, mean: 283.2 μm^3^; *P* < 0.0001 and 13.99 μm; *P* < 0.0001; CSF, mean: 459.8 μm^3^; *P* < 0.001 and 14.52 μm; *P* < 0.05) and Rec1 (FBS, mean 257.2 μm^3^; *P* < 0.0001 and 14.28 μm; *P* < 0.0001; CSF, mean: 316.2 μm^3^; *P* < 0.0001 and 13.32 μm; *P* < 0.0001) strains. H99 and Rec1 cells exposed to FBS or CSF show no differences in cell volume (**Figure 8B**) whereas H99 cells evinced higher capsule size than Rec1 cells only after incubation with CSF (**Figure 8D**). In FBS, the cell body size of Rec1 (mean: 6.88 μm) was significantly larger than those observed in H99 (mean: 6.34 μm; *P* < 0.0001) and *plb1* (mean: 6.14 μm; *P* < 0.0001) strains (**Figure 8C**). H99 and *plb1* cells show no differences in cell body size after FBS induction. All the strains grown with CSF exhibited substantial reduction in cell body size relative to Sabouraud or FBS, but no difference in cell body size was observed in the CSF between them (**Figure 8C**). These findings indicate that FBS and human CSF stimulate *plb1* cell capsular enlargement, suggesting that PLB1 disruption alters cryptococcal cell capsular production regulation during peripheral or CNS infection.

### *plb1* and Rec1 cryptococci show altered cell wall, and this impacts their CPS stability and secretion ability

**Figure 9:**
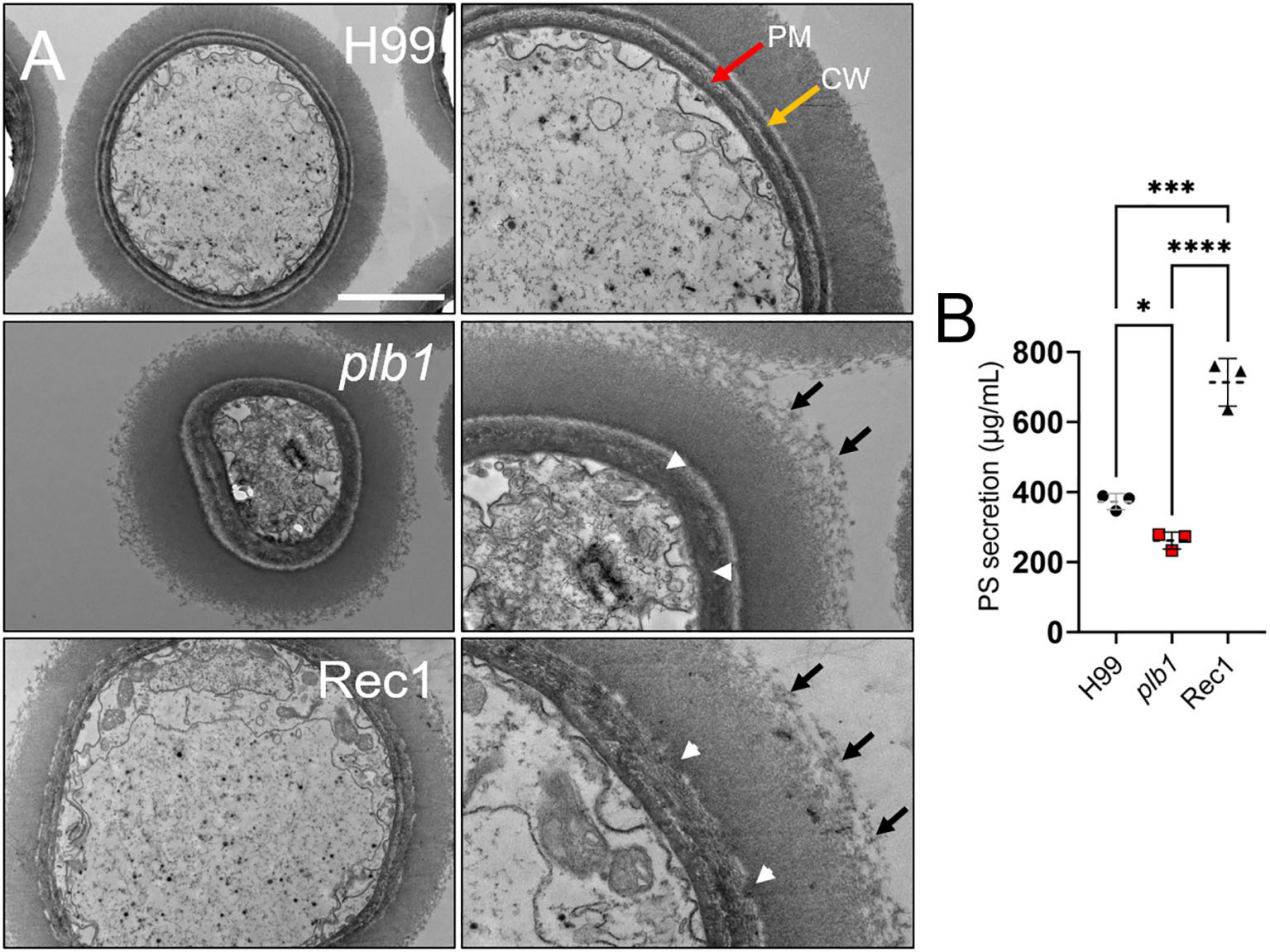
*Cn* strains *plb1* and Rec1 exhibit capsular surface and cell wall alterations relative to H99. **(A)** Transmission electron microscopy (TEM) images of *Cn* strain H99, *plb1*, and Rec1 cells. PM (red arrow) and CW (yellow arrow) denote plasma membrane and cell wall, respectively. Black arrows indicate polysaccharide fibrils extending (middle row, right panel) and detaching (bottom row, right panel) from the capsule. Scale bars: 2 µm (left panels) and 1 µm (right panels). White arrowheads denote cell wall structural alterations for *plb1* and Rec1 strains. **(B)** Polysaccharide (PS) secretion from each strain (*n* = 3 independent cultures and measurement per strain) was quantified in the supernatant of a 24 h culture in Sabouraud dextrose broth using the Dubois method. Dashed lines indicate the averages of the results, and error bars denote SDs. Asterisks denote *P* value significance (****, *P* < 0.0001; ***, *P* < 0.001; *, *P* < 0.05) calculated by ANOVA and adjusted using Tukey’s *post hoc* analysis.

*Cn* CPS secretion is critical for fungal survival during infection (36) and CNS invasion (37). We used transmission electron microscopy (TEM) to examine morphological changes on the cell wall and polysaccharide capsule of H99, *plb1*, and Rec1 and their impact on capsular secretion (**Figure 9**). H99 cells displayed a thin well-defined cell wall and a uniform and compact capsule with smooth edges at the tip of the polysaccharide fibers (**Figure 9A**; upper panels). In contrast, *plb1* cells revealed a thicker and disorganized (white arrowheads) cell membrane merged with the cell wall as well as visible polysaccharide fibers (black arrows) protruding at the edge of the capsule (**Figure 9A**; middle panels). Rec1 cells also showed a thicker and disordered cell membrane fusing with a structurally defective (white arrowheads) cell wall that lacks a clear line of separation to distinguish between the cell wall and capsule interface (**Figure 9A**; lower panels). Moreover, the edge of Rec1 cell capsules present a lack of delineation in the edges and loose polysaccharide material (black arrows).

Given the structural differences observed in the regions near the cell wall and edges of each *Cn* strain, we compared and quantified polysaccharide secretion in 7-d cultures (*n* = 3 per group) of H99, *plb1*, and Rec1. *Cn* Rec1 cells released the highest levels of CPS relative to H99 (*P* < 0.001) and *plb1* (*P* < 0.0001; **Figure 9B**), suggesting that Rec1 synthesizes CPS but may exhibit impaired CPS anchoring to the cell wall, resulting in increased CPS shedding *in vivo* and *in vitro*. H99 cryptococci also showed significantly higher CPS secretion than *plb1* cells (*P* < 0.05). Our results suggest that *plb1* and Rec1 have a defective cell wall that affects CPS anchoring and release.

### *Cn PLB1* disruption reduces capsular elasticity

**Figure 10:**
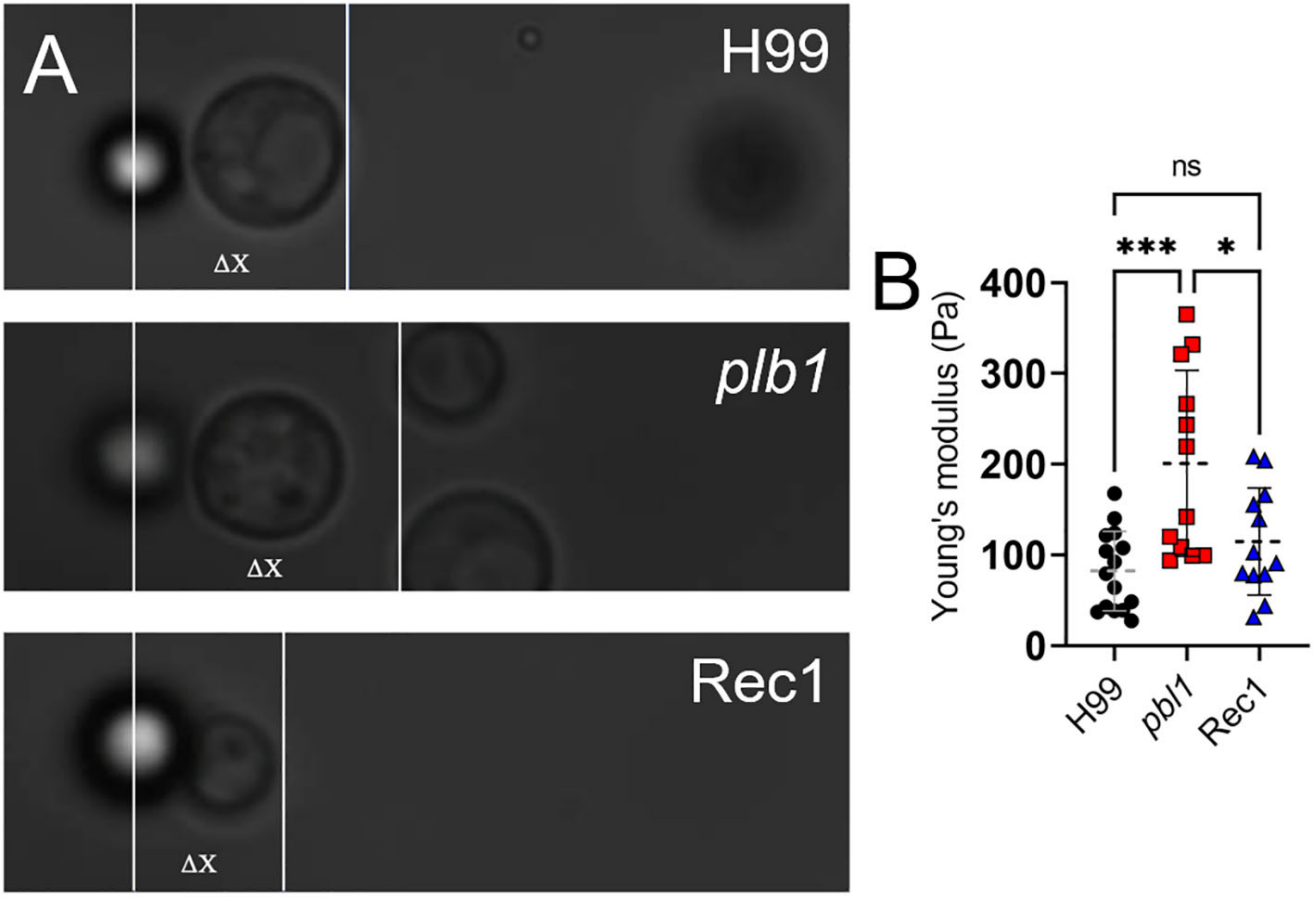
*Cn plb1* capsular PS demonstrated a higher average Young’s or elastic modulus value than H99 and Rec1 strains. **(A)** Representative bright field microscopy images of single *Cn* H99, *plb1*, and Rec 1 cells attached to a bead used to measure capsular deformation and calculate Young’s modulus as a quantification of PS elasticity. ΔX represents the displacement of the cell from its initial position while remaining in contact with the stationary bead. **(B)** Values of Young’s modulus for *Cn* H99, *plb1*, and Rec1 capsular PS. Each symbol represents individual measurements per strain (*n* = 12). Dashed lines indicate the averages of Young’s modulus values calculated for individual cryptococci (of each strain) using an optical tweezer, and error bars denote SDs. Asterisks denote *P* value significance (***, *P* < 0.001; *, *P* < 0.05; ns, not significant) calculated by ANOVA and adjusted using Tukey’s *post hoc* analysis.

Capsule elasticity is associated with increased pathogenicity during cryptococcal infection, particularly in fungal systemic dissemination and CNS invasion (38). To evaluate the influence of PLB1 on capsular fiber elasticity, the mechanical response of the capsule was assessed using an optical tweezer setup by first attaching a *Cn* cell from H99, *plb1*, and Rec1 strains to stationary polystyrene beads and then pulling the cell in the opposite direction (**Figure 10A**). Cell displacement (ΔX) was measured until detachment from the bead, providing a relative comparison of fibril length and elasticity. The Young’s or elastic modulus of the capsule was calculated for cryptococci of each strain (*n* = 12–14 cells). The Young’s modulus was significantly increased in *plb1* (mean: 200.9 Pa; range: 93.7 to 365.2 Pa) compared with H99 (mean: 82.4 Pa; range: 27.5 to 167.9 Pa; *P* < 0.001) and Rec1 (mean: 114.9 Pa; range: 31.6 to 208.5 Pa; *P* < 0.05), which inversely correlates with reduced capsular fiber elasticity (**Figure 10B**). There were no statistical differences when *Cn* H99 and Rec1 strains were compared. These results support the importance of PLB1 in influencing the structural integrity and flexibility of *Cn* CPS, prompting further investigation into this critical virulence factor and its involvement in infection.

## Discussion

Biofilm formation contributes to *Cn* resistance to antifungal drugs (39), host molecules (40), and phagocytic cell responses (31). Cryptococcomas are biofilm-like lesions formed in tissue during invasive *Cn* infection, particularly in pulmonary (41) and cerebral (2, 42, 43) infections. PLB1 activity has been shown to promote adhesion of *Cn* to human lung epithelial cells (18), initializing invasive processes including hematogenous dissemination (20) and traversal of the BBB (44), resulting in cerebral cryptococcosis. We recently revealed the importance of PLB1 in *Cn* colonization of the CNS and establishment of CME (21), but the implications of PLB1 in cryptococcoma formation have not been fully elucidated. In this study, we investigated the involvement of PLB1 in *Cn* biofilm-like lesion formation using a recently described stereotaxic i.c. mouse model of infection (22).

*plb1*- and Rec1-i.c. infected mice demonstrated considerably delayed mortality relative H99-infected mice, likely due to reduced fungal proliferation and ability to form cryptococcomas in the basal ganglia, the brain region of infection. The unexpected behavior of Rec1 and its dissimilarity to H99 present an important limitation of our study, however its use in examining PLB1 and potential off-target effects of genetic manipulation provide insight into the complexity of PLB1 in *Cn* virulence. Secretory PLB1 activity can hydrolyze phospholipids of host cell membranes, contributing to neuronal damage (45), which may explain the strong microglial response observed in H99- and Rec1-infected tissue. Interestingly, PLB1 secretion induces the synthesis of host eicosanoids, which are regulators of host immunity, neuroprotection, and cerebral blood flow (16, 46, 47). In the lungs, PLB1 promotes eicosanoid production during cryptococcal infection (16), which is associated with decreased inflammation (20), a plausible explanation to the significant difference observed in microglial recruitment in brain tissues infected with H99 and Rec1 7-dpi. This effect has not been described in the context of CME, however prostaglandin E2 has been associated with BBB disruption in bacterial meningitis (48), suggesting a similar potential mechanism of PLB1 action in response to tissue destruction. We previously showed that systemically (e.g., intravenous)-infected mice with H99 or Rec1 had similar brain tissue colonization behavior and mortality (21), and all three strains, including *plb1*, demonstrated increased fungal burden in the brain from 3- to 7-dpi following i.v. infection. In contrast, i.c.-infected mice with Rec1 unexpectedly demonstrated similar cerebral cryptococcosis progression and mortality as animals infected with the *plb1* strain. It is possible that Rec1 or optimal PLB1 activity is stimulated in circulation or following exposure to serum or immune factors prior to invading the CNS, which may explain the reduced virulence relative to the wild-type H99 strain observed in the i.c. model of infection.

The egg yolk emulsion assay is a widely-described qualitative and semi-quantitative assay used to assess PLB1 activity, particularly in *Candida albicans* (49), as well as in multiple environmental and clinical *Cn* strains (50). The results of the egg yolk assay confirm the phenotype of *PLB1* disruption, revealing absence of precolonial precipitate around *plb1* growth, and complement the genomic results illustrating *PLB1* disruption in the mutant and gene-reconstituted strains. However, as part of the *PLB1* promoter remains in the disruption construct of *plb1*, it is possible that truncated forms of *PLB1* may contribute to our observations, despite a phenotype distinct from H99 and Rec1 being produced. Our findings raised interesting questions about the insertion sites of the *PLB1* gene within the reconstituted genome and the genetic regulatory factors of *PLB1* expression that may have impaired the ability of the Rec1 strain to effectively control *PLB1* expression during *in vivo* infection, resulting in the inability of the Rec1 strain to form a cryptococcoma upon direct inoculation into the CNS. Thus, whole-genome sequencing for H99, *plb1*, and Rec1 strains demonstrated a disruption of *SGF29* in both the mutant and reconstituted strains, but not H99. Mutations in this gene result in reduced histone acetylation and may promote hypervirulence, as observed in clinical strains (23). Also, Rec1 exhibits enhanced GXM secretion *in vivo*, in the intravenous (systemic (21)) and i.c. (this study) models of infection, and hyperactivity or increased cell body size, at least in serum (**Figure 8C**), potentially due to dysregulation of this gene. The hyperactivity of Rec1 may be dependent on the presence of the *PLB1* gene, which could be why the *plb1* strain is deficient in virulence compared with Rec1. While the potential disruption of *SGF29* is a possible explanation for differences between Rec1 and H99, the contribution of this gene is beyond the scope of our investigation but is important to explore in future studies through the generation of multiple independent complemented mutant strains.

*Cn* infection control seems to be related to the number of phagocytic microglia present near the cryptococcoma area. *plb1*-infected tissue shows between 30-40% phagocytic cell presence in or around the biofilm-like lesions. Similarly, the brain of these animals had 50-60% ramified or branched microglia, suggesting minimal disturbance of tissue homeostasis. The presence of high number of apparently healthy ramified or homeostatic microglia in brains infected with the defective PLB1 mutant correlates with and validates our previous findings using the systemic model (21). The higher proportions of phagocytic microglia in brain tissue of *plb1*-infected mice correlate with our previous *in vitro* observations of enhanced phagocytosis and killing of *plb1* cryptococci by NR-9460 microglia-like cells (21), suggesting that PLB1 confers resistance to microglial effector functions. H99- and Rec1-infected brains evinced extensive abundance of dystrophic microglia, which has been linked with neurodegenerative diseases such as Alzheimer’s, Parkinson’s, and multiple sclerosis (51). In fact, the observation that phagocytic and ramified microglia were replaced by dystrophic cells in H99-infected tissue from 3- to 7-dpi suggests that the dystrophic phenotype is related to the progression of cerebral cryptococcosis and increased host mortality. A similar trend was observed in tissue removed from Rec1-infected brains. It is conceivable that PLB1 may enable GXM dissemination, as seen in H99- and Rec1-infected tissue, which may be a trigger for microglia to adopt this phenotype in response to antigenic stimulation or exposure. Further investigation is needed to discern whether the reduction in cryptococcoma formation by *plb1* and Rec1 strains results from reduced virulence or containment of the infection by neuroprotective immune responses. We recently demonstrated that active production and secretion of the capsular material altered the morphology and distribution of microglia around cryptococcomas (52). Hence, in future studies, we can isolate capsular and secreted GXM from each of these strains to determine their structural composition and understand the importance or potential involvement of PLB1 in capsular production and secretion, respectively. We could expose microglia-like cells to each strain’s GXM and elucidate their impact on these CNS resident cell morphology and effector functions (e.g., phagocytosis, chemotaxis, nitric oxide production, etc.).

The polysaccharide capsule confers virulence to *Cn* through a variety of functions including reduced leukocyte migration and phagocytosis. Our studies demonstrated that *plb1* cells induce significantly higher capsule size in human CSF relative to H99 and Rec1 cells. This is not surprising considering that FBS has been previously shown to stimulate *plb1* capsular enlargement (53) and confirmed in our studies (**Figure 8**). Similarly, *plb1* cells have shown higher capsular enlargement inside of J774 macrophages after 18 h phagocytosis than H99 and Rec1 cells (54). Since capsular size enlargement was observed by H99 and Rec1 cells after culture with CSF, although not to the extent shown by *plb1* cells, this suggests that cryptococcal capsule growth can be modulated or repressed by PLB1. In this regard, the activity of PLB1 has been suggested to repress titan cell formation during infection (54). In contrast, we found that GXM is substantially released in tissue infected with H99 or Rec1 strains. Our results confirmed the results obtained in the systemic model of infection that Rec1 cells secrete more CPS in tissue especially surrounding the cryptococcoma than H99 cells. Importantly, *Cn* can release GXM into the microenvironment during the formation of biofilms for immune protection (55) and structural matrix stability (29). In *plb1-*infected mice, GXM intensity was reduced compared with animals of the other groups, suggesting that the ablation of PLB1 secretion may hinder CPS release or other secretory pathways. Interestingly, Rec1 had the highest intensity of GXM dissemination *in vivo* and secreted the highest concentration of CPS *in vitro.* These observations suggest a connection between the secretory pathways of PLB1 and GXM, as disruption and reconstitution of the *PLB1* gene may have resulted in the reduction and dysregulation of CPS secretion by *plb1* and Rec1, respectively.

*Cn* active GXM production and extracellular CPS secretion are necessary for biofilm formation (30). Confocal microscopy demonstrated that *plb1* and Rec1 strains formed thinner biofilms than the H99 strain. Nevertheless, H99- and Rec1-biofilms exhibited higher metabolic activity and higher biofilm-derived cells than *plb1*-biofilms. *Cn* biofilm formation involves fungal adherence to cellular surface, proliferation, and production of an extracellular polysaccharide matrix that provides structural integrity and protection (30). *Cn* biofilm maturation is correlated with thickness and metabolic activity (30). The inability of *plb1* cells to form biofilms suggests that PLB1 deficiency facilitates capsule enlargement, given that their cryptococci have larger capsules in induction conditions. Nevertheless, *plb1* cells show structural capsular defects characterized by protruding CPS fibers at the edges and reduced CPS fiber elasticity and CPS secretion. These observations, in addition to *plb1* cell low metabolic activity and viability, may critically affect their ability to form biofilms *in vitro* and cryptococcomas *in vivo*. Moreover, *plb1* cryptococci showed impaired adhesion to SH-SY5Y neuroblastoma cells and decreased cell CPS fiber (SEM) contact or local cell-derived GXM secretion to a solid surface (ELISA spot). The reduced thickness observed in *plb1* and Rec1 biofilms compared to H99 biofilms may be related to the structural alterations found in the cell wall, which may also affect the attachment and composition of the polysaccharide capsule, which is essential for biofilm formation (30). Furthermore, inside of macrophages, PLB1 localizes at the neck of newly forming buds in H99 cryptococci (56). This suggests that PLB1 may have a role in bud development and cryptococcal replication, and thus it is possible that the inability of the *plb1* cells to properly bud would also explain the biofilm formation defect observed (54). Future studies using live microscopy and comparing biofilm formation by these cryptococcal strains are necessary to validate this hypothesis.

While Rec1 cryptococcomas displayed the largest GXM release *in vivo* and *in vitro*, it is possible that structural modifications in the capsule or CPS compromises the aggregation of the carbohydrate fibers around the cryptococci, regardless of having similar cellular density or growth rate as H99 biofilms. This hypothesis is supported by SEM and TEM images showing that Rec1 cells have smaller capsules and shorter CPS fibers than H99 cells, disordered cell wall-capsule interface, and loose CPS material at the edge of the capsule. Although Rec1 cells showed similar capsule size when grown in induction conditions, and the CPS fibers demonstrated similar elasticity to H99 cells, potential structural defects in the cell wall due to genetic manipulation may result in difficulty for the cell to anchor and retain its CPS. This complication is observed most evidently near the capsule, which could explain this strain having the most extensive CPS released *in vivo* and *in vitro* and the highest CPS fibril array anisotropy. In fact, having the largest cell body after capsular induction conditions indicates that Rec1 cells’ high metabolic activity may be associated with CPS synthesis and high secretion to compensate for the reduced ability of these cell-derived CPS to adhere locally to solid surfaces, demonstrated in the ELISA spot assay. Besides, even though Rec1 cells bound better than *plb1* cells to neuroblastoma cells, binding was similarly observed into brain cells (**Figure 5A**; yellow arrows) and solid surfaces (white arrows), which explain why there was no difference in binding in the ELISA spot assay (**Figure 6C**). Indeed, it is possible that in addition to the capsule, the presence of a yet unidentified adhesin explains the strong binding of *plb1* and Rec1 to the plastic surface.

PLB1 is localized in the cell wall, anchored to the cell membrane by GPI proteins (14). Both *plb1* and Rec1 cells have a disorganized and thicker plasma membrane that fuses with the cell wall. A previous study has shown that PLB1 provides structural support to the cell wall during heat stress because *plb1* cells show compromised cell wall integrity (57). The dramatic alterations in the *plb1* plasma membrane suggest PLB1 is critical for cell wall formation. Similarly, genetic reconstitution of PLB1 resulted in Rec1 cells having potential defects in the cell wall, particularly in the cell wall-capsule interface, which may affect PLB1 or CPS anchoring to the cell membrane. PLB1 is attached to β-1,6-glucans present in the cell wall that comprise the mobile matrix of the cell wall due to their high hydration levels compared to other *Cn* cell wall constituents (58). As secreted PLB1 contains β-1,6-glucan (57), it is plausible that the cell wall structure may become altered by the absence or dysregulated secretion of PLB1 in the *plb1* and Rec1 strains, respectively, due to a potential change in cell wall β-1,6-glucan proportions, however this possibility has not been explored. Although further studies are necessary, both genetically manipulated strains also displayed altered CPS at the edge of their capsules, indicating that PLB1 may also be implicated in the vesicular transport of CPS through the cell wall (59) and regulates its secretion into the environment (14).

Although neither is a natural route of cryptococcal infection, it is important to differentiate the progression of cerebral cryptococcosis in C57BL/6 mice after i.c. (stereotaxic CNS) and i.v. (systemic) inoculation. The main advantage of the i.v. model of cryptococcal infection is that it mimics how *Cn* transmigrates from the bloodstream to the CNS via interactions with the BBB. Additionally, it allows the fungus to interact with peripheral immunity that can influence CNS responses prior to fungal invasion. In contrast, stereotaxic injections are extensively used in neuroscience and are the safest method to study CNS-related infectious disease including cryptococcosis (60–63). In fact, the i.c. infection model allows us to directly study cerebral cryptococcosis progression using a controlled inoculum. We selected the i.c. infection model over the systemic model due to the resulting focal localization of cryptococcomas in the mouse brain that resembles human lesions. Human cerebral cryptococcosis shows meningoencephalitis with (cryptococcoma) and without (no cryptococcoma) involvement of the brain parenchyma, especially in immunosuppressed and immunocompetent individuals, respectively (64). This can also be replicated in the i.c. model depending on the site of infection. For instance, direct inoculation in the basal ganglia or cortex and ventricles results in progression of cerebral cryptococcosis with and without parenchyma involvement, respectively (22). We also reported that i.c. H99-infected mice show similar CME manifestations as humans (64, 65) including edema and intracranial pressure (8–10 dpi) and hydrocephalus (>12 dpi), making it excellent for studying neurotoxic impact of *Cn* infection, GXM release, and disease progression (22). Pulmonary and systemic infections often result in a high number of cryptococcomas with wide brain dissemination, compromising our ability to study the impact of *Cn* infection on specific brain regions and their resulting cognitive/motor impacts. Nevertheless, despite the disadvantages of each of these mouse models of *Cn* infection, they can also be used individually or combined to address fundamental questions to understand the pathogenesis of cerebral cryptococcosis.

For more than a decade, the field of bacteriology has accepted that the major hallmarks of *in vivo* biofilms are thus aggregated bacteria, which tolerate the host defense and high concentrations of antimicrobial agents over time (66). *Cn* not only forms cryptococcomas in the brain of immunocompromised individuals optimally treated with antifungal drugs, but also these fungal aggregates are surrounded by an extensive polysaccharide matrix, the classical definition of biofilms (67), and are impervious to elimination by CNS-resident microglia (52), providing clear evidence to recognize these structures as true *in vivo* biofilms. In fact, cryptococcomas in the brain parenchyma often require surgical resection for patient recovery (43, 68). The nature of cryptococcoma formation or parenchymal involvement in the brain is poorly understood. It is unclear the role of the initial attachment stage that precedes cellular mass formation in the brain parenchyma. We demonstrated that adhesion to neuroblastoma cells by each cryptococcal strain was associated with their ability to form biofilms. In addition, it is well documented *Cn*’s ability to form biofilm *in vitro* (69) and on medical devices implanted in patients with cryptococcosis with (28) or without (70) CNS involvement. In contrast, microbial biofilm aggregates can form without attachment to a surface (71, 72). It is plausible that cryptococci do not adhere to brain tissue but still form cryptococcomas with cells that show biofilm-related features including increased metabolic activity, immune cell and molecular tolerance, and antifungal resistance.

## Conclusion

**Figure 11:**
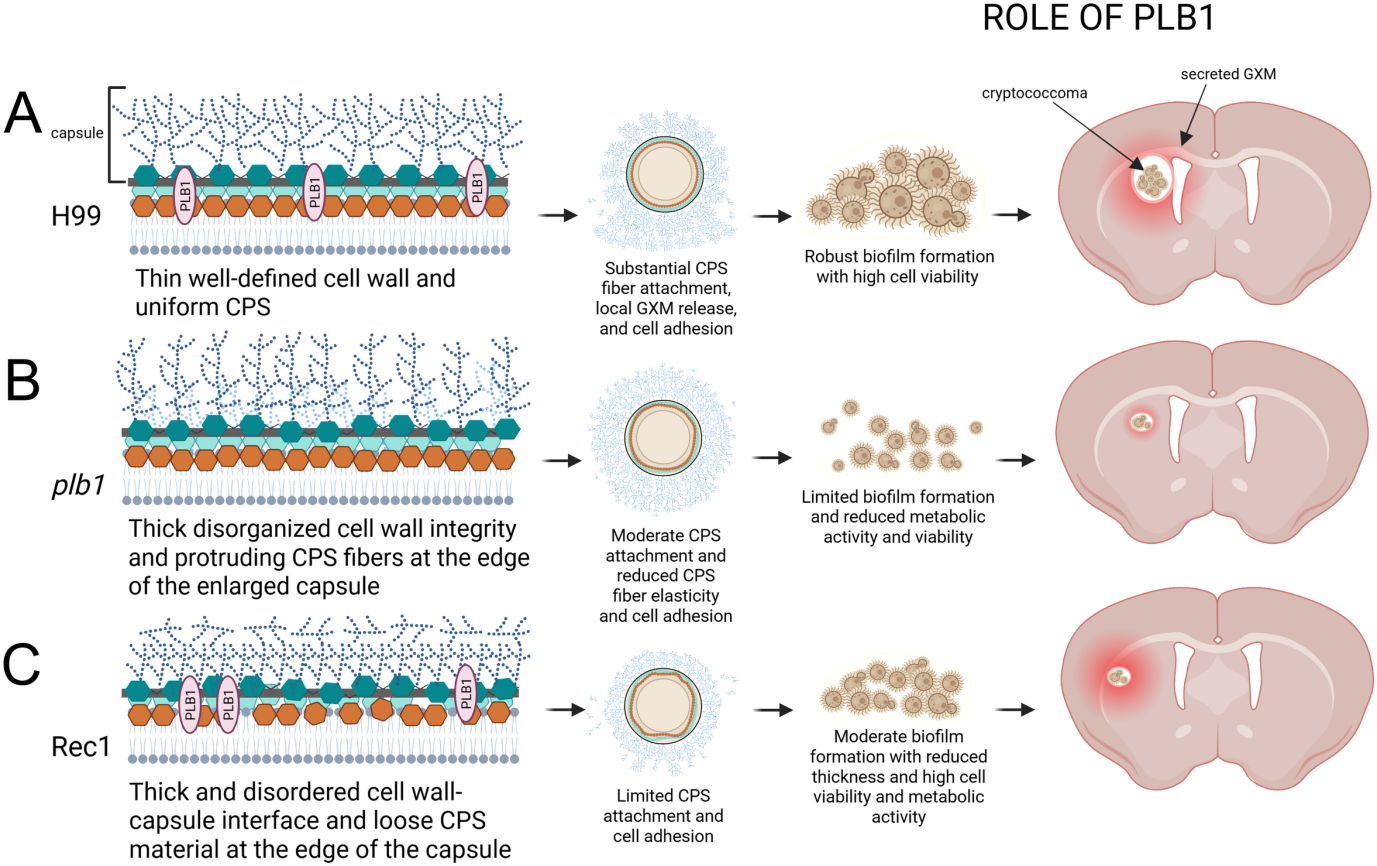
Model of *Cn* PLB1 involvement in biofilm-like cryptococcoma formation and brain parenchyma colonization. PLB1 is associated to the cryptococcal cell wall. **(A)** *Cn* H99 cells have a symmetrical plasma membrane and cell wall organization, PLB1 distribution, and polysaccharide capsule. H99 cells secrete capsular polysaccharide (CPS) extensively facilitating their adhesion to tissue, localized proliferation, and cryptococcoma formation, which is characterized by a biofilm-like structure consisting of cryptococci surrounded by abundant amounts of exopolysaccharide. **(B)** Cryptococci lacking PLB1 have a thicker plasma membrane fused to the cell wall. The capsule near the cell wall shows high polysaccharide density whereas distinguishable polysaccharide fibers are observed at the tip of the enlarged capsule. CPS release is compromised in *plb1* cells affecting tissue adhesion and cryptococcoma formation. **(C)** Rec1 cells lack delineation of the cell wall-capsule interface. Defects in the cell wall region attached to the capsule are visible, which can compromise the anchoring of the polysaccharide fibers to the cell wall, promoting substantial polysaccharide secretion. In this sense, Rec1 cells also display loose threads of polysaccharide at the tip of the capsule. Moreover, Rec1 cryptococci evince considerable reduction in the local release or adhesion of the CPS, which may affect cryptococcoma formation. The proposed model was created by Melissa E Munzen with BioRender.com.

We reveal that cryptococcoma formation and *Cn* brain colonization is dependent on the function of PLB1. This enzyme is required for cryptococcal adhesion to neuron-like cells or tissue by stimulating local CPS production, release, and extracellular accumulation resulting in cryptococcoma formation (**Figure 11**). PLB1 is critical for cell plasma membrane, cell wall, and capsular stability. PLB1 deficiency makes *Cn* susceptible to elimination by microglial cells, indicating its importance in modulating brain tissue immune response. It is necessary to highlight that our finding that Rec1 has cell envelope structural alterations is significant and does not diminish the importance of this or *plb1* strain for the field. Systematic investigation of this strain provides a multitude of studies that can be performed to understand how these structural modifications are important for pathogenesis or regulation or location of this enzyme in the cell wall in relationship to the capsule and extracellular vesicles production and secretion, as well as resistance to antifungal drugs. Finally, these results support the validity of PLB1 as a target for future antifungal therapeutics in combination with other antifungal strategies to prevent tissue destruction caused by cryptococcoma formation or treatment to promote host immunity, which may be important for the prevention, treatment, and management of CME.

## Materials and methods

### Cn

*Cn* isogenic strains H99, *plb1*, and Rec1 were kindly provided by John Perfect at Duke University and another pair of these strains were provided by Arturo Casadevall at Johns Hopkins Bloomberg School of Public Health for result confirmations. *plb1* is a PLB1 mutant and a ura5 auxotroph of H99 with a single insertion transformed by using biolistic DNA delivery with a knockout construct containing URA5 inserted into PLB1 (15). Rec1, or reconstitution of the *plb1* strain, was performed by transforming the mutant strain with a construct containing the entire plb1 gene and the selectable antibiotic resistance gene HygB (73). *plb1* and Rec1 exhibit similar growth rates, melanin production, and capsule sizes to H99 (15). Rec1 also shows similar phospholipase synthesis and secretion relative to H99 (15). Yeasts were grown in Sabouraud dextrose broth (pH 5.6) (BD Biosciences) for 24 h at 30 °C in an orbital shaker (New Brunswick Scientific) set at 150 rpm (nominal to early stationary phase). Growth in neuroblastoma complete and Sabouraud dextrose broth was assessed in real-time at an optical density (OD) of 600 nm every 2 h using a microplate reader (Bioscreen C; Growth Curves USA).

### i.c. *Cn* infection

C57BL/6 female mice (6–8 weeks old; Envigo) were anesthetized using isoflurane (3-5% for induction and 1-2% maintenance; model: VetFlo Vaporizer Single Channel Anesthesia System, Kent Scientific), placed in prone position over a heating pad (model: RightTemp Jr., Kent Scientific), and prepped using standard aseptic techniques. A local anesthetic, bupivacaine or ropivacaine (0.05%; Covetrus), was administered subcutaneously in the incision. The fur on the skull was carefully shaved off and the animal was securely placed in a stereotaxic apparatus (model: 940; Kopf Instruments). Using a small hand-held microdrill (model: Ideal microdrill; Braintree Scientific), the skull was thinned until the underlying dura mater was visible and a 26 G Hamilton syringe was brought to the correct stereotaxic position and lowered until it touched the exposed dura. The craniotomy was around 1 mm in diameter and the correct brain coordinates were identified using a stereotaxic brain atlas (e.g., The Allen Mouse Brain Atlas; https://mouse.brain-map.org/static/atlas). Based on our recent study (22) and others (74, 75) using the *Cn* H99 strain, a 1-μL suspension containing 10^4^ cryptococci in sterile saline was injected into the striatum [Stereotaxic coordinates: x (medial/lateral), -2; y (anterior/posterior), 0.2; z (dorsal/ventral), -3.5)] using a 26 G Hamilton syringe connected to a pump (model: UltraMicroPump3; World Precision Instruments). We injected the fungal inoculum in a 1-μL volume to avoid tissue damage or diffusion of the cryptococci to other regions of the brain. The skin incision on the dorsal head was closed with sterile nylon suture and 2-4% topical chlorhexidine solution was applied over the closed incision. After the surgery, the mice were placed in a clean recovery cage and monitored for survivability. The survival end points were inactivity, tachypnea, or loss of ≥ 25% of body weight from baseline weight. We monitored the mice twice daily for clinical signs, dehydration, and weight loss. Animals showing signs of dehydration or that lost more than 10% weight received supportive care such as 1 mL of parenteral fluid supplementation (saline), and moist chow on the cage floor was provided. In separate infections, brain tissues were excised for processing for determination of CFU numbers and histopathological studies.

### CFU determinations

Brains were excised from euthanized mice and weighed 3- and 7-dpi. The brain tissue was homogenized in 5 mL of sterile phosphate buffered saline, serially diluted, and a 100 μL suspension was plated on Sabouraud dextrose agar (BD Difco) and incubated at 30 °C for 48 h. Quantification of viable yeast cells from infected animals were determined by CFU counting of two dilutions per animal (*n* = 12 per day) and the results were normalized per gram of tissue.

### Brain histology

The brains were harvested and immersed in 4% paraformaldehyde (Fisher) overnight. Then, brains were washed three times (3X) with sterile saline for 1 h, embedded in paraffin, 4 μm coronal sections were serially cut using a microtome, fixed onto glass slides, and subjected to hematoxylin & eosin (H&E) staining to examine tissue morphology. GXM (mAb 18B7 is an anti-cryptococcal GXM IgG1 generated and generously provided by Arturo Casadevall at the Johns Hopkins Bloomberg School of Public Health; 1:1,000 dilution) and Iba-1 (rabbit anti-human Iba-1; 1:1,000 dilution; FujiFilm Wako) specific Ab (conjugated to horseradish peroxidase; dilution: 1:1,000; Santa Cruz Biotechnology) immunostaining to assess capsular release/distribution and microglial number/phenotype, respectively, near cryptococcomas. The slides were visualized using a Leica DMi8 inverted microscope, and images were captured with a Leica DFC7000 digital camera using LAS X digital imaging software. GXM intensity distribution in tissue sections at 10X magnification (*n* = 10 fields per brain; each field was 1,920 x 1,440 pixels or 1,244 x 933 μm) was calculated using the National Institutes of Health (NIH) ImageJ bundled with 64-bit Java 8 color deconvolution tool software (version 1.53t). The mean color intensity of the GXM for each treatment group was plotted in Prism 10.3.1. (GraphPad). The images were examined and analyzed by Dr. Mohamed F. Hamed, a veterinary pathologist.

### Whole-genome sequencing

The genomic DNA extraction was performed using the NucleoSpin DNA Yeast kit (Macherey-Nagel). Isolated DNA was eluted and stored at -20 °C until shipment to Azenta Life Sciences (New Jersey) for Next Generation sequencing (protocol adapted from Azenta Life Sicences). For the library preparation, genomic DNA was quantified using the Qubit 2.0 Fluorometer (ThermoFisher Scientific). NEBNext^®^ Ultra™ DNA Library Prep Kit for Illumina, clustering, and sequencing reagents was used throughout the process following the manufacturer’s recommendations. Briefly, the genomic DNA was fragmented by acoustic shearing with a Covaris S220 instrument. Fragmented DNA was cleaned up and end repaired. Adapters were ligated after adenylation of the 3’ends followed by enrichment by limited cycle PCR. DNA libraries were validated using a High Sensitivity D1000 ScreenTape on the Agilent TapeStation (Agilent Technologies) and were quantified using Qubit 2.0 Fluorometer. The DNA libraries were also quantified by real time PCR (Applied Biosystems). The sequencing library was clustered onto a flowcell and loaded onto the Illumina Novaseq according to manufacturer’s instructions. The samples were sequenced using a 2 x 150bp Paired End (PE) configuration. Libraries were sequenced for a minimum of 12 million reads on the Illumina NovaSeq X Plus platform using 150 base PE sequencing. FASTQ files were trimmed using Cutadapt (v. 5.0) to trim Illumina adaptors (i.e. AGATCGGAAGAGCACACGTCTGAACTCCAGTCA R1 adaptor sequence; AGATCGGAAGAGCGTCGTGTAGGGAAAGAGTGT R2 adaptor sequence), with minimum read length 35 bp and low-quality bases with a quality phred-like score < 30. Reads < 30 pair bases were excluded from DNA genome analysis. Burrows-Wheeler Alignment tool was used to map FASTQ files to the *Cn* var. grubii H99 (RefSeq; GCF_000149245.1_CNA3) reference genome. Aligned genome sam files were converted to bam files, sorted, and indexed before visualization in Integrative Genome Viewer (IGV, v. 2.18.2).

### Egg yolk emulsion assay

*Cn* strains were screened for extracellular phospholipase production by the method of Chen et al. (50) with minor modifications. In brief, the egg yolk medium contained Sabouraud dextrose agar (BD Biosciences) with 1 M sodium chloride, 0.005 M calcium chloride, and 10% sterile egg yolk. The egg yolk was prepared from eggs sterilized for 1 h in 70% ethanol (Thermo Fisher), followed by aseptic separation of the yolk. The egg yolk was directly added to the sterilized supplemented medium and allowed to solidify in agar plates (Thermo Fisher). Strains were washed twice in PBS and diluted to a concentration of 10^7^ cryptococci/mL, and a 5 µL volume of each strain was added to the cooled agar plates for incubation for 7 days. Phospholipase activity was determined by presence of precipitation or halo zone surrounding cryptococcal growth. Precipitation zone (Pz) was measured using FIJI ImageJ and calculated as Pz = (DC + PZ)/DC; DC = Diameter of Colony, PZ = Precipitation Zone. Each strain was evaluated in duplicate colonies. The mean Pz value was calculated and plotted in Prism 10.3.1. (GraphPad Software).

### *Cn* biofilm formation

*Cn* cells were suspended at 10^7^ cells per mL in minimal medium (20 mg/mL thiamine, 30 mM glucose, 26 mM glycine, 20 mM MgSO_4_ · 7H_2_O, and 58.8 mM KH_2_PO_4_; pH 5.5; Sigma). For each strain, 100 μL of the suspension were added into 900 μL of fresh minimal medium in each individual well of polystyrene 6-well plates (Corning) and incubated at 37 °C. Biofilms were formed over 48 h. Following the adhesion stage, the wells containing *Cn* biofilms were gently washed 3X with PBS to remove non-adhered cryptococci using a multichannel pipette. Measurement of biofilm formation and viability was done by assessing the metabolic activity of the attached cells with the 2, 3-bis (2-methoxy-4-nitro-5-sulfophenyl)-5-[(phenylamino) carbonyl]-2H-tetrazolium-hydroxide (XTT; Sigma) reduction assay and CFU determinations (29).

### Confocal microscopy

*Cn* biofilms were incubated for 45 min in 75 μL of PBS containing the fluorescent stain FUN-1 (10 μM; Molecular Probes). Then, wells were blocked with PBS (1% BSA). GXM-specific mAb 18B7 (2 μg/mL) was added, and the plate was incubated. Fluorescein isothiocyanate (FITC; Molecular Probes)-conjugated goat anti-mouse (GAM)-IgG1 at 1 μg/mL in PBS (1% BSA) was applied. Between steps, the wells were washed with 0.05% Tween 20 (Thermo Fisher) in Tris-buffered saline (TBS; Thermo Fisher). All incubations were done at 37 °C for 1 h. FUN-1 (Excitation wavelength, 470 nm; emission, 590 nm) is converted to orange-red cylindrical intravacuolar structures by metabolically active cells, while mAb 18B7, when bound by FITC-conjugated GAM IgG1 (excitation wavelength, 488 nm; emission, 530 nm), labels GXM and fluoresces green. Microscopic examinations of biofilms formed in glass-bottom plates were performed with confocal microscopy. Depth measurements across the width of the device were taken at regular intervals using an upright Leica TCS SP5 confocal laser scanning microscope (Leica). To determine the structure of the biofilms, a series of horizontal (*X*-*Y*) optical sections with a thickness of 1.175 μm were taken throughout the full length of the biofilm. Confocal images of green (18B7-FITC) and red (FUN-1) fluorescence were recorded simultaneously using a multichannel mode. *Z*-stack images and measurements were corrected utilizing the Leica LAS X software in the deconvolution mode.

### Fungal adhesion to human SH-SY5Y neuroblastoma cell line

The human SH-SY5Y neuroblastoma cell line was used in this study. These cells are derived from a metastatic bone tumor from a 4-year-old cancer patient (ATCC) and are widely used for studying physiology and pathology of neurodegenerative diseases (76). These cells grow as a mixture of floating and adherent cells. The cells grow as clusters of neuroblastic cells with multiple, short, fine cell processes. SH-SY5Y cells were cultured in complete medium (1:1 mixture of Eagle’s Minimum Essential and HAM’s F12 media) supplemented with 10% heat-inactivated FBS (Bio-Techne), 100 U/mL penicillin (Gibco), and 100 µg/mL streptomycin (Gibco). Exponentially growing cells, at approximately 60-75% confluence, were counted, and seeded into 96-well microtiter plates (Corning). The incubation conditions of the cells were 37 °C in a humidified 5% CO_2_ atmosphere. For adhesion experiment, 10^5^ neuroblastoma cells were cultured with 10^6^ H99, *plb1*, or Rec1 cells for 2 h at 37 °C in 5% CO_2_ and washed 3X with PBS. Then, fluorescent microscopy was performed to visualize fungal adhesion.

### Fluorescent microscopy

Monolayers of SH-SY5Y cells were fixed on sterile circular glass coverslips (18 mm; Thermo Fisher) with 3% paraformaldehyde (Thermo Fisher) in PBS for 10 min at room temperature (RT). The cells were washed one time with PBS-GSA (1X PBS containing 10 mM glycine and 0.2% sodium azide; Thermo Fisher) to remove the fixative and quench the reaction. The cells were permeabilized for 3 min with freshly made PBS-GSA containing 0.5% Triton X-100 (Sigma). Then, washed one time with PBS-GSA and blocked with 1% bovine serum albumin in PBS-GSA for 5 min at RT. After blocking, the cells were again washed 3X with 5% Tween 20 in PBS and incubated with anti-β tubulin (neuroblastoma cell body; rabbit polyclonal; 1:50 dilution; Proteintech) conjugated to Alexa Fluor 488 (green; 1:200 dilution; Invitrogen) and anti-GXM (1:200 dilution; 18B7) conjugated to Alexa Fluor 555 (red; 1:200 dilution; Invitrogen), respectively, in blocking solution in an orbital shaker (New Brunswick Scientific) at 150 rpm and 37 °C for 1 h. The samples were washed 3X with blocking buffer and incubated with 4′,6-diamidino-2-phenylindole (DAPI; blue; Thermo Fisher) to stain neuroblastoma nuclei for 1 h at 37 °C. The slides were washed 3X with PBS, coverslips were affixed, and each sample was viewed to determine the adhesion of cryptococci to the surface of SH-SY5Y cells (*n* = 100) with a Zeiss LSM 700 Confocal Laser Scanning Microscope at a magnification of 40X. Images were collected using an AxioCam digital camera and analyzed using Zen Lite digital imaging software.

### SEM

Cryptococci were washed 3X in PBS (pH 7.3) and fixed in a 2.5% glutaraldehyde solution grade EM (Electron Microscopy Sciences) prepared in sodium cacodylate buffer 0.1 M (pH 7.2) for 45 min at RT. Then, the cells were washed 3X in 0.1 M sodium cacodylate buffer (pH 7.2) containing 0.2 M sucrose and 2 mM MgCl_2_ (Merck Millipore), and adhered to coverslips thickness N°.1 (0.13 - 0.17 mm), Ø12 mm (Waldemar Knittel Glasbearbeitungs GmbH) previously coated with 0.01% poly-L-lysine (Sigma) for 20 min. Adhered cells were then gradually dehydrated in an ethanol growing series 30, 50 and 70% for 5 min and 95% and 100% twice for 10 min (Merck Millipore). The coverslips were then critical-point-dried using a Leica EM CPD300 critical point drier and mounted on pin stub specimen mounts Ø 12.7 mm x 8 mm (TedPella, Inc.) using a PELCO Tab Carbon Conductive (TedPella, Inc.). Following this, the samples were coated through sputter coating with a 10 nm layer of platinum using the Q150R Plus (Quorum Technologies). The samples were observed using a Carl Zeiss Evo LS microscope and images were collected with their respective software packages.

### ELISA spot

Cryptococci were counted and diluted to a concentration of 10^4^ cells per mL in minimal medium as described previously (30). For each strain, a 100 μL suspension was added in duplicates to individual wells of a polystyrene 96-well plate and cryptococci were allowed to adhere for 2 h at 37°C. Following the adhesion stage, the wells were washed 3X with 0.05% Tween 20 in TBS to remove non-adhered cryptococcal cells using a microtiter plate washer. The wells were then blocked for non-specific binding for 1 h at 37 °C, by adding 200 μL of 1% BSA in PBS. Next, 2 μg/mL of GXM binding mAb 18B7 in PBS (1% BSA) was incubated overnight at 4 °C. Then, 1 μg of biotin-labeled goat anti-mouse IgG1/mL was added for 1 h at 37°C. Between every step, the wells were washed with 0.05% Tween 20 in TBS. After the biotinylated mAb step, a 50 μL of Vectastain (Vector Laboratories) was added to each well and incubated for 30 min at RT. Wells were washed and incubated in 50 μL of 3, 3’- diaminobenzidine prepared in an equal volume of hydrogen peroxide and diluted in PBS for 10–15 min before being washed twice in tap water and air dried. Each well was visualized using 20X and 100X objectives of a Leica DMi8 inverted microscope, and spots were counted after images were captured with a Leica DFC7000 digital camera using LAS X digital imaging software. Spot area was analyzed in six 20X power fields using FIJI ImageJ and calculated as Area = πr^2^. Each strain was evaluated in duplicate wells.

### Anisotropy determination

Quantification of CPS fiber anisotropy was performed using FibrilTool (32), an ImageJ plug-in that determines the average orientation of a fiber array. The anisotropy value ranges from a maximum of 1, when all fibers point in the same direction (perfectly ordered), to a minimum of 0, when fibers are randomly oriented (no order).

### Capsule induction conditions

To induce capsule growth, 10^7^ *Cn* cells per mL were inoculated in 6-well plates with 2 mL of PBS supplemented with 10% FBS or human CSF and incubated overnight without agitation at 37°C (77). Fungi grown in Sabouraud dextrose broth alone were used as a negative control. To measure capsule size, ten-microliter aliquots were spotted on microscope slides and mixed with India ink (Gibco). Each slide was visualized using a Leica DMi8 inverted microscope at a magnification of 100×. All images were captured with a Leica DFC7000T digital camera using the Leica digital imaging software platform LAS X. To calculate the capsule volume, the diameter of the whole cell and the cell body were each measured with LAS X digital imaging software and capsule volume was defined as the difference between the volume of the whole cell (yeast cell + capsule) and the volume of the cell body (as limited by the cell wall). Volumes were calculated using the equation for volume of a sphere as 4/3πr^3^. Between 55 and 90 cells per strain were measured and cell volume, cell body size, and capsule size were reported.

### TEM

Cryptococci were washed 3X in PBS and subsequently fixed in a 2.5% (v/v) glutaraldehyde solution grade in 0.1 M sodium cacodylate buffer and microwaved (350 watts, 3 pulses of 30 sec each with an interval of 60 sec between pulses). Subsequently, the cells were washed 3X in 0.1 M sodium cacodylate buffer and fixed using 1% (v/v) osmium tetroxide (OsO_4_), 0.8% (v/v) potassium ferrocyanide and 5 mM calcium chloride, in 0.1 M cacodylate buffer (pH 7.2) for 10 min, washed twice in water, and then incubated in 1% (w/v) thiocarbohydrazide in water, for 5 min (all reagents from Electron Microscopy Sciences). After three washes in water, cells were again incubated in the fixative osmium solution for 2 min and finally washed 3X in water. Next, cells were gradually dehydrated in an acetone (Merck Millipore) series: 50%, 70%, 90%, and two subsequent 100%. All the dehydration procedures were performed in the microwave (350 watts, 10 sec pulses for each step). The Spurr resin (Electron Microscopy Sciences) was gradually used to substitute acetone in the following proportions: Spurr (v:v): 3:1, 2:1, 1:1, 1:2, 1:3 and finally pure Spurr. Each mixture was also submitted to the same microwave cycles for 2 min except the last step (pure Spurr) that was performed without any radiation. The polymerization step was carried out for 48 h in an oven at 70 °C. The samples were sliced in ultrathin sections, 75-nm were obtained with a Leica C7 ultramicrotome, collected formvar‐coated copper slot grids, and submitted to an incubation with 5% (w/v) uranyl acetate in water for 20 min and lead citrate for 5 min for contrasting. Finally, the samples were observed in a TEM model HT7800 *RuliTEM* (Hitachi High-Tech) equipped with an AMT CMOS 8.192K camera.

### Quantification of secreted CPS in culture using the phenol-sulfuric acid method

A cell density of 10^7^ *Cn*/mL for each strain was cultured in minimal medium for 7-d at 37 °C under constant agitation (100 rpm). The yeasts were removed by centrifugation (~6,600 *g*) in a conical tube, and the supernatant was analyzed as described by Dubois *et al.*, 1956 (78), with a final volume of 10 mL using: I) pure supernatant, II) 7.5 mL of supernatant + 2.5 mL of pyrogen-free water, III) 5 mL of supernatant + 5 mL of pyrogen-free water, IV) 2.5 mL of supernatant + 7.5 mL of pyrogen-free water.

### Young’s modulus measurement

Glass-bottom dishes were coated with 10 μg*/*mL of mAb 18B7, and 200-μL suspensions of 10^4^ *Cn* cells in PBS were added to the plates and then incubated for 1 h at RT. After washing with PBS to remove nonadherent cells, polystyrene beads were added to the plate and the samples were placed in the optical tweezer (OT) system. To measure the Young’s modulus of the PS capsule of *Cn* H99, *plb1*, and Rec1 strains, a polystyrene bead (*a* = 1.52 ± 0.02 μm) was first captured with the OT and then pressed against the capsule for 1 min, to attach the bead to the fungus capsule. The microscope stage was then moved with a controlled velocity (*V* = 0.071 ± 0.002 μm/s). As a result, the attached bead changed its equilibrium position in the trap and the fungal capsule deformed. Images of the entire process were captured using a CCD Hamamatsu C2400 camera and, using ImageJ software, the change in equilibrium position of the trapped bead, ΔX, was determined as a function of time. With this result the Young’s modulus was determined.

### Statistical analysis

All data were subjected to statistical analysis using Prism 10.3.1 (GraphPad Software). Differences in survival rates were analyzed by the log rank (Mantel-Cox) test. *P* values for multiple comparisons were calculated by one-way analysis of variance (ANOVA) and were adjusted by the use of the Tukey’s *post hoc* analysis. *P* values of < 0.05 were considered significant.

## Data Availability

All data generated or analyzed during this study are included in this published article. Requests to access the datasets should be directed to LMartinez@dental.ufl.edu.
